# Synthesis and Biological Evaluation of New Schiff Bases Derived from 4-Amino-5-(3-fluorophenyl)-1,2,4-triazole-3-thione

**DOI:** 10.3390/molecules28062718

**Published:** 2023-03-17

**Authors:** Sara Janowska, Dmytro Khylyuk, Michał Janowski, Urszula Kosikowska, Paulina Strzyga-Łach, Marta Struga, Monika Wujec

**Affiliations:** 1 Department of Organic Chemistry, Faculty of Pharmacy, Medical University, 4a Chodzki Str., 20-093 Lublin, Poland; 2 Department of Pharmaceutical Microbiology, Faculty of Pharmacy, Medical University, 1 Chodzki Str., 20-093 Lublin, Poland; 3 Chair and Department of Biochemistry, Faculty of Medicine, Banacha Str. 1, 02-097 Warsaw, Poland

**Keywords:** synthesis, Schiff bases, antimicrobial activity, antifungal activity, cytotoxicity, docking studies

## Abstract

The treatment of infectious diseases is a challenging issue faced by the medical community. The emergence of drug-resistant strains of bacteria and fungi is a major concern. Researchers and medical professionals are working to develop new and innovative treatments for infectious diseases. Schiff bases are one a promising class of compounds. In this work, new derivatives were obtained of the 4-amino-5-(3-fluorophenyl)-2,4-dihydro-3*H*-1,2,4-triazole-3-thione reaction, with corresponding benzaldehydes with various substituents at position 4. The antibacterial and antifungal activities of all synthesized compounds were tested. Several new substances have shown moderate antifungal activity against *Candida* spp. The highest activity directed against *C. albicans* was shown by compound RO4, with a 4-methoxyphenyl moiety and an MIC value of 62.5 µg/mL. In order to check the toxicity of the synthesized compounds, their effect on cell lines was examined. Additionally, we tried to elucidate the mechanism of the antibacterial and antifungal activity of the tested compounds using molecular docking to topoisomerase IV, D-Alanyl-D-Alanine Ligase, and dihydrofolate reductase.

## 1. Introduction

The treatment of infectious diseases remains an important and difficult issue due to the accumulation of many problems related to therapy, such as the development of resistance to currently known drugs and the very rapid increase in opportunistic fungal and bacterial infections in immunocompromised patients (e.g., AIDS, cancer) during chemotherapy or after organ transplantation [1,2,3]. Since the emergence and growth of antimicrobial resistance is faster than the rate of discovery and introduction of new effective medicines, the shortage of effective drugs for pathogen eradication and combating diseases is a global problem at present. Large problems are posed by opportunistic microorganisms, including both bacteria and fungi, especially those with a low infective potency but resistance towards known antimicrobials. Despite the existence of many groups of compounds with antimicrobial activity, there is still an increase in the number of strains of bacteria and fungi that are resistant to these substances [1]. Therefore, there is a growing need to create new classes of drugs with different mechanisms of action than those that were previously known. Moreover, they may be novel antimicrobials, especially for the treatment of infections caused by multi-drug resistance strains such as methicillin-resistant staphylococci, especially the pathogenic *Staphylococcus aureus* (MRSA). The development of such therapeutics would reduce the risk of cross-resistance with currently existing drugs [4]. There is an urgent need to develop compounds directed against new therapeutic targets [5,6,7]. New directions of development of antibiotics present an optimized workflow for the discovery of new agents with activity against bacterial transcription or replication and growth [7]. A potential approach to block or reduce the growing resistance is to inhibit the target bacterial proteins, which are important or critical to basic microbial life processes, e.g., translation, nucleic acid replication, or cell wall biosynthesis, by novel compounds [8]. Only a few new antibiotics have been approved in the last decade to combat the emergence of multidrug-resistant bacterial strains [6]. They include new compounds or their combination, such as eravacycline (tetracycline derivatives), delafloxacin (fourth-generation fluoroquinolones), meropenem and vaborbactam (one β-lactam and one β-lactamase inhibitor combination), cefiderocol (cephalosporin), plazomicin (aminoglycoside), and pretomanid, which are being developed to treat adults with drug-resistant (both multi-drug and extensively drug-resistant) tuberculosis [6]. This serious global problem is related to the rapid increase in the number of infections with high-priority opportunistic pathogen etiology, which are mainly bacterial, including MRSA and coagulase-negative staphylococci (CoNS), *Campylobacter* spp. or *Escherichia coli*, *K. pneumoniae*, Salmonella spp., *Shigella* spp., and other carbapenem-resistant *Enterobacteriaceae*. A promising new line of antimicrobial research is the use of polymeric materials, nanostructure systems, and nano-particles, as well as combined therapies [6].

Heterocyclic rings are one of the most important elements of the structure of many antibacterial drugs available on the market [9]. The incorporation of the 1,2,4-triazole system into the molecule may affect the lipophilicity of the molecule and its ability to form hydrogen bonds. The use of the heterocyclic ring of 1,2,4-triazole in the design of drugs can improve the pharmacological and pharmacokinetic parameters or reduce the toxicity of compounds [4]. The 1,2,4-triazole heterocyclic system is present in many molecules showing therapeutic potential in tests [10], including analgesic [11], antibacterial [10,12,13], antifungal [10,14], anti-inflammatory [15], anticancer [16,17] and anti-tuberculosis activity [18]. In addition, drugs belonging to the group of 1,2,4-triazoles, such as antiviral Ribavirin, anti-migraine Rizatriptan, and anti-anxiety Alprazolam [10], are used in modern medical practice.

One promising direction for the search for new drugs among 1,2,4-triazole derivatives is compounds with antifungal activity, particularly focusing on species such as *Candida albicans* and *Candida tropicalis*. This activity was confirmed in many in vitro tests [6,10]. Antifungal drugs such as Fluconazole, Posaconazole, and Intraconazole are the best examples of highly active molecules with a triazole system [10]. In recent years, scientific reports on the promising antifungal activity of compounds from the Schiff base group have also been published [19,20,21]. 

Schiff bases are a group of compounds containing an azomethine group (-HC=N-) and showing a wide range of biological activity [22]. Structurally, Schiff bases are nitrogen analogs of aldehydes and ketones, in which carbonyl groups have been replaced by azomethine or imino groups [23,24]. Substances containing this structure have been shown to have antifungal, antibacterial, antiviral, antimalarial, cytostatic, anti-inflammatory, and antipyretic properties [23,25]. The azomethine group can be found in the structures of several drugs currently used in medicine, such as nifuroxazide and thiacetazone, which are oral antibacterial agents [26]. Due to this range of activities and synthesis methods that allow for the easy multidirectional modification of Schiff base structures, compounds from this group are a promising area for searches for new drugs [27,28]. In recent years, compounds from the Schiff base group have been intensively studied as potential antibacterial agents [26,28,29,30,31,32,33]. There are reports of high activity in compounds containing an azomethine moiety against multidrug-resistant bacterial strains, e.g., *S. aureus* (especially methicillin-resistant *S. aureus*, MRSA) or *Staphylococcus epidermidis*, including reports of stronger effects than reference drugs such as ciprofloxacin [26,34]. Compounds from the group of Schiff bases also exhibited a higher activity than the reference fluconazole against multidrug-resistant pathogenic fungi classified as *Candida* [34,35,36].

Based on the above literature data, we decided to design and synthesize a series of compounds that are new derivatives of 1,2,4-triazole with the character of Schiff bases, derived from 4-amino-5-(3-fluorophenyl)-2,4-dihydro-3*H*-1,2,4-triazole-3-thione. The aim of developing this new series of compounds was to find new antimicrobial drugs that are effectivene against strains of bacteria and fungi that cause difficult-to-treat nosocomial infections, such as *S. aureus*, *C. albicans*, and *C. tropicalis*. The antimicrobial activity of the obtained compounds was determined as the MIC and MBC values using the broth microdilution method against a panel of reference strains of bacteria and fungi. We tried to elucidate the mechanism of action of the molecules using in silico tests based on molecular docking.

## 2. Results and Discussion

### 2.1. Chemistry

**Scheme 1:**
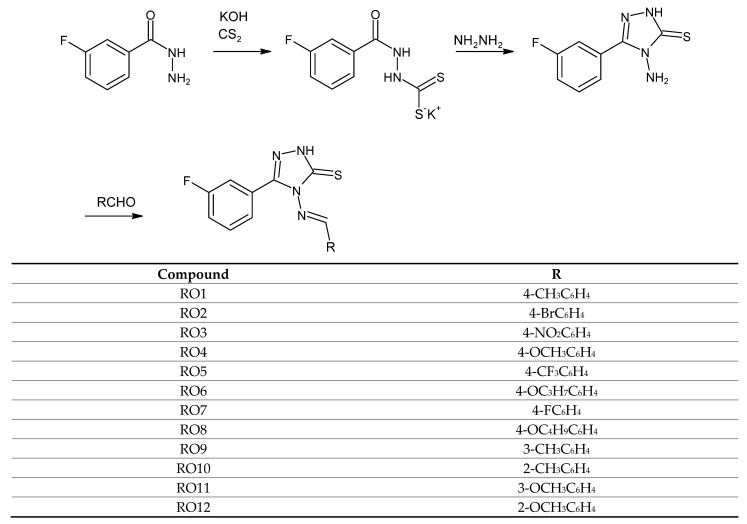
Synthesis of Schiff bases of 1,2,4-triazole-3-thione derivatives.

The synthesis of new compounds from the group of Schiff bases was carried out according to the synthetic route shown in Scheme 1.

The initial RO1-RO8 series consisted of compounds that are reaction products of 4-amino-5-(3-fluorophenyl)-2,4-dihydro-3*H*-1,2,4-triazole-3-thione and corresponding benzaldehydes with various substituents at position 4. The purpose of this combination of molecules was to determine the effect that the type of substituent has on the antimicrobial activity in this group of compounds. We then expanded the series of compounds due to the distinctive activity of molecules with a methyl (RO1) and methoxy (RO4) group against *C. albicans*, accompanied by trace antibacterial activity. To better understand the observed relationship, we synthesized compounds with -CH_3_ and -OCH_3_ moieties at positions 2 and 3. The enlargement of the series of compounds aimed to determine the effect of the substituent’s position on the aromatic ring on its antibacterial and antifungal activity.

In the first stage, the reaction of 3-fluorobenzoic acid hydrazide with carbon disulfide in a potassium base medium provided the potassium salt of 3-fluorophenyldithiocarbazinic acid. Subsequently, the reaction with hydrazine hydrate provided 4-amino-5-(3-fluorophenyl)-2,4-dihydro-3*H*-1,2,4-triazole-3-thione. The yield of this step in the synthetic pathway was 80%. The aminotriazole obtained was converted into Schiff bases through a reaction with aromatic aldehydes, which differed regarding the type and position of these substituents on the ring. These compounds were obtained in high yields in the range of 53–95%. Ten of the planned series of twelve compounds were obtained. Six of the compounds were new, previously undescribed derivatives of 1,2,4-triazole-3-thione. The synthesis of two of the planned compounds, i.e., one with 4-bromobenzaldehyde and the other with 4-trifluoromethylbenzaldehyde, failed, despite the many attempts that were made.

As mentioned above, the synthesis process yielded six new molecules that had not been previously described in the scientific literature: RO3, RO6, RO8, RO10, RO11, and RO12. Compounds RO1, RO4, and RO9 had been previously described in the scientific literature, but there were no data on the physicochemical properties of these molecules (^13^C NMR, ^1^H NMR, and IR spectra), and their antimicrobial activity had not been previously evaluated. RO7 had been previously described in terms of both physicochemical data and antimicrobial activity; however, we found the methodology to be objectionable [37].

The structures of the new compounds were determined using IR, ^1^H NMR, ^13^C NMR spectroscopy, and elemental analysis. The ^1^H NMR spectra showed chemical shifts in the protons related to atoms N2, which confirmed the formation of cyclic 1,2,4-triazole compounds. The signal from the hydrogen atom on the nitrogen atom of the triazole ring was visible in the range of 14.29–14.44 ppm. The analysis of ^1^H NMR nuclear magnetic resonance spectra indicated the presence of characteristic signals for the protons of the =CH group in the range of 9.49–10.07 ppm and the hydrogens of aromatic rings in the range of 7.05–8.40 ppm.

The ^13^C NMR spectra of the carbons of compounds RO1, RO9, and RO10 showed carbon signals resulting from the resonance of the methyl group in the range of 19.47–21.76 ppm, while compounds RO4, RO6, RO8, RO11, and RO12 showed signals indicating the presence of the methoxy group in the range of 56.07–69.86 ppm. Signals for other carbon atoms of the synthesized molecules were observed at the expected chemical shift values.

IR spectroscopy showed the presence of an N=CH bond, characteristic of Schiff bases, in the range of 1593–1607 cm^−1^. 

The ^1^H NMR (Figures S1–S10) and ^13^C NMR (Figures S11–S20) spectra for all new compounds are presented in the Supplementary Materials.

### 2.2. Antimicrobial Evaluation

The in vitro antimicrobial activity of the synthesized 1,2,4-triazole-3-thione Schiff base derivatives was tested based on the inhibition of the growth of reference strains of bacteria and fungi in vitro using the broth microdilution method. The antibacterial and antifungal results (MIC and MBC/MFC value) are reported in Table 1 and Table 2. 

The standard antibiotic ciprofloxacin and cefuroxime against bacteria were used as a positive control of antibacterial activity detection. The MIC values for these antibiotics were 0.24–0.98 µg/mL and 0.24–62.5 µg/mL, respectively. Nystatin was used as a positive control against yeast reference strains. Nystatin with MIC = MBC = 0.24–0.48 µg/mL was used as a positive control for antifungal activity. 

Generally, the highest susceptibilities of Gram-positive opportunistic bacteria to the tested R04-R010 compounds were exhibited by staphylococci–coagulase-positive *S. aureus* ATCC 6538 with an MIC range from 125 to 1000 µg/mL and coagulase-negative *S. epidermidis* ATCC 12228 with an MIC range from 250 to 1000 µg/mL. The MICs of the R04-R09 Schiff bases towards the *M. luteus* ATCC 10240 strain were in the range of 125–250 µg/mL.

All the tested compounds showed no or minimal activity against reference strains of Gram-negative oxidase-negative bacteria *K. pneumoniae* ATCC 13883 and *E. coli* ATCC 25922 with MIC ≥ 1000 µg/mL. The highest activity against these bacteria was exhibited by the RO9 compound with the MIC values in the range of 125–250 µg/mL. All the tested compounds did not show any activity against oxidase-positive *P. aeruginosa* ATCC 27853 bacteria (MIC ≥ 1000 µg/mL).

In the analysis of the data collected in Table 1, attention should be paid to the high selectivity of the newly synthesized Schiff bases. Although the antimicrobial activity of the molecules is not very high, they are selective towards a narrow group of opportunistic bacterial and fungal pathogens. For example, the RO12 molecule showed antifungal activity against almost all the reference strains of *Candida* spp. yeast used in the investigation, including high activity against *C. albicans* ATCC 10231 (MIC = 125 µg/mL), with no antibacterial activity. However, RO9 showed promising activity against *S. aureus* and *S. epidermidis* staphylococci, which can be responsible for difficult-to-treat nosocomial infections, but it showed no antimicrobial activity against Gram-negative bacteria and yeasts. The high degree of selectivity of the substance action is a promising feature in the context of the development of modern antifungal and antibacterial drugs.

The novel Schiff bases synthesized in the present study showed compound- and concentration-dependent activity against selected species of the reference Gram-positive bacteria, including the opportunistic pathogen *S. aureus*. Our compounds exhibited high MIC values towards Gram-negative bacteria. 

Gram-positive bacteria are common and important nosocomial opportunistic pathogens. Infections caused by these organisms are of great concern due to the poor health status of people, their increased incidence, and the high level of multidrug resistance. As indicated by our results, some of the tested Schiff bases showed moderate potential antibacterial activity against selected reference strains of Gram-positive bacteria, including *S. aureus* ATCC 6538, *S. epidermidis* ATCC 12228, *B. subtilis* ATCC 6633, and *M. luteus* ATCC 10240. The MIC values determining the antibacterial potential of the newly synthesized compounds were higher than the values for the reference drugs. The strongest activity towards *S. aureus* ATCC 6538, with an MIC level of 125 µg/mL, was shown by two compounds: RO9, with a 3-methylphenyl substituent, and RO10, with a 2-methylphenyl substituent. Together with the previously undescribed RO8 molecule with a 4-butoxyphenyl substituent, RO9 had the highest activity against *S. epidermidis* ATCC 12228, with an MIC value of 250 µg/mL. It can be seen that the highest antibacterial activity against staphylococci was exhibited by compounds with low-polarity substituents on the aromatic ring (methyl and butoxy groups). The RO8 and RO9 molecules with the highest activity against both species of staphylococci, *S. aureus* and *S. epidermidis,* had no or trace activity against the Gram-negative bacterial strains and yeasts used in the test. This indicates the high selectivity of the antimicrobial activity of the RO8 and RO9 molecules. The new RO10 molecule, which was previously not described in the scientific literature, exhibited selective activity against the reference strain of *S. aureus* with an MIC of 125 µg/mL, while showing no significant activity against the other reference strains of bacteria used in the test. 

The tested compounds exhibited antifungal activity against various reference species of *Candida* spp. Three types of methods have been standardized by standard development organizations: broth dilution, disk diffusion, and azole agar screening for fungi [38]. It is very important for diagnostics to consider not only the determination method but also the interpretation of in vitro data. The antimicrobial activity of the Schiff base assigned as RO7 during our examination was published earlier by Aouad [37]. As reported by the author, compound RO7 was characterized by high activity against the tested strains of bacteria (MICs = 16–125 µg/mL) and *C. albicans* (MIC = 4 µg/mL). However, we included this compound in our investigation to make it coherent. In contrast to the results reported by Aouad [37], the R07 compound analyzed in our study did not show antibacterial and antifungal activity with high MIC or MFC values, respectively. These discrepancies may be related to the different origins of the microbial species or strains and the different study methodologies, including broth microdilution techniques. The most important differences were related to the use of different bacterial strains in the study. Moreover, we used the EUCAST (2022) methods, which differ from the methods used by Aouad [37]. Besides, we did not use Sabouraud broth to determine antifungal activity toward the tested *Candida* spp. yeasts during our investigation. This medium is not suitable according to the recommendations for the determination of MFC values, as well as other methods for the detection of these compounds, e.g., the agar well or disc diffusion method for fungi, both yeasts (e.g., *Candida* spp.) or dermatophytes (e.g., *Trichophyton* spp.), and molds (e.g., *Aspergillus* spp.) For these determinations, the most popular method is using a synthetic RPMI medium with MOPS buffer or Mueller–Hinton agar, or broth medium supplemented with glucose to a final concentration of 2%. At this concentration, for the medium ensures suitable fungal growth [38,39,40]. The methods used by Aouad [37] and those applied in the present study differed in terms of the DMSO solvent distribution. During our study, this solvent was used only to prepare the stock concentration (50 mg/mL). During the preparation of the starting concentrations of the tested compounds (1000 µg/mL), the broth medium was used, which completely eliminated the possible effects of DMSO at high concentrations. 

*Candida* species, especially *C. albicans*, are the most common and widely known etiological factor of all types of candidiases, including hospital-acquired infections associated with high mortality [41,42]. Other emerging non-*albicans Candida* spp. (NAC), e.g., *C. glabrata*, *C. krusei*, or *C. parapsilosis* also pose serious nosocomial threats [43,44]. The number of clinically available antifungal drugs has increased in recent years. Among the current commercially available antimycotics used for the treatment of candidiasis, three major classes are known: azoles (e.g., fluconazole or posaconazole), echinocandins (e.g., caspofungin or micafungin), and polyenes (e.g., amphotericin B or nystatin) [42,43]. Their antifungal mechanisms and influence on fungal cells differ. Polyenes lead to lysis of the fungal cell membrane by binding ergosterol, while azoles, with their fungistatic activity, inhibit ergosterol biosynthesis. The different effect of the echinocandin class is related to its possibility of blocking fungal cell wall synthesis in the (1,3)-*β*-D-glucan part, exhibiting a fungicidal effect. Moreover, candidiases are difficult to treat due to their fungal resistance to many antimycotics, especially azoles, used against biofilm-forming cells [41,44,45]. 

The number of antifungal susceptibility tests performed during research investigations and in laboratories has increased in recent years. The goal of an antifungal investigation is to reliably determine the inhibitory concentration values that may be used in standard patient therapy and to track the rates of fungal resistance against drugs. In recent decades, the number of people with risk factors for fungal diseases, including invasive infections, has dramatically increased. In addition, acquired resistance has emerged in fungal species such as *C. glabrata*, *C. auris*, and *Aspergillus fumigatus*. 

*Candida* spp. yeasts are opportunistic pathogens that can cause diseases of various parts of the body in patients with exposure to long courses of broad-spectrum antibiotics, implanted medical devices, reduced immunity, or disturbed homeostasis of natural microbiota and in elderly people. Recently, there has been a need to search for new antifungals due to the dramatically increased occurrence of *Candida* spp. opportunistic infections (candidiasis). These fungal infections affect patients undergoing chemotherapy, with HIV infection, or taking immunosuppressive drugs after transplantation. Invasive fungal infections (IFIs) caused by yeasts are the most serious type of infection of the brain, heart, blood, or other organs [46,47]. Such infections pose a major therapeutic challenge, and their treatment is often ineffective due to the low sensitivity or resistance of yeasts to the known and currently used antimycotics. Another important issue related to the therapy for infections caused by yeasts is the disturbance of the protective function of the patient’s microbiota, which normally prevents the uncontrolled development of fungal pathogens [48]. As shown by our results, some of the tested compounds showed selective activity towards various *Candida* species, including *C. albicans* and *C. glabrata*. These Schiff bases, characterized by their outstanding antifungal activity, can also show species-dependent antibacterial activity. This relationship is exceedingly beneficial because *C. albicans* infections are accompanied by losses in the patient’s bacterial microbiota, and a drug with antibacterial properties can further increase the scale of this problem. The highest activity against *C. albicans* in the tests was shown by compound RO4, with a 4-methoxyphenyl moiety with an MIC value of 62.5 µg/mL, which indicates a promising antifungal effect. Another outstanding compound in terms of activity against *C. albicans* with an MIC value of 125 µg/mL is RO12, with a 2-methoxyphenyl substituent. The activity against this yeast strain was also shown by RO1, with a 4-methylphenyl group (MIC = 250 µg/mL). As indicated by the data collected in Table 1, the anti-yeast activity of the tested compounds from the group of Schiff bases is affected by both the type and position of the substituent attached to the aromatic ring at position 4 in relation to the 1,2,4-triazole system. Small electron donor substituents, such as the methoxy and methyl groups, are elements of the structure of molecules that have a positive effect on their anti-yeast properties. At the same time, this activity depends on the position of these substituents in the aromatic ring: the *para* position is associated with the highest activity, the *ortho* position with medium activity, and the *meta* position with the lowest activity against *C. albicans*.

In the tests, RO12 showed the highest anti-yeast activity among all the new molecules that had not been previously described in the scientific literature. In addition to the activity against *C. albicans*, it also showed moderate antifungal potential against the reference strains of *C. auris*, *C. lusitaniae*, and *C. tropicalis,* with an MIC value of 500 µg/mL. At the same time, this compound did not show any activity against all the reference strains of bacteria used in the tests. The MIC values obtained for the molecule against the aforementioned *Candida* strains are not very low but, given the high selectivity (lack of activity against bacteria) and the low cytotoxicity against normal human cell lines, this compound seems to have a promising therapeutic potential.

The minimum bactericidal/fungicidal concentration (MBC/MFC) was determined for compounds with MIC ≤ 500 µg/mL for bacteria and yeasts. These data indicate that the MBCs of the tested compounds typically do not exceed their respective MICs by a factor of more than 1–4 times. The fungicidal activity was observed only in the case of RO11 (methoxy group in the *meta* position) against *C. albicans*, with MIC = 500 µg/mL and MFC = 1000 µg/mL. Among the tested Schiff bases, we showed no fungicidal activity of the R04 compound with the highest MIC value and no activity towards *C. albicans* in the tested range of compound concentrations (MFC > 1000 µg/mL). This suggests that the tested compound is fungistatic rather than fungicidal.

### 2.3. Cytotoxic Evaluation

In vitro tests on normal and neoplastic cell lines showed very the low cytotoxic potential of the tested compounds. All the tested molecules achieved IC_50_ scores above 100 µM against MDA-MB-231 and PC3 tumor cell lines. All the tested triazole derivatives also showed very low cytotoxicity against normal cell lines (Table 3). With the exception of RO12, for which the IC_50_ could be accurately calculated and was 98.08 µM (38.09 µg/mL), all the tested compounds showed an IC_50_ in the range above 100 µM. The obtained data indicate the low toxicity of the synthesized triazole derivatives to human cells.

The data from the cytotoxicity measurements performed using human healthy and cancer cells, combined with the results of antibacterial and antifungal activity, suggest the highly selective biological effects of the new triazole derivatives.

### 2.4. Docking

The docking research suggests that the new compounds have multiple potential mechanisms of antibacterial activity. The results of the docking to the MBL are rather contradictory. Most compounds demonstrated binding energies that were higher than the reference ligand, and there were no correlations between the obtained scores and biological activities. Nevertheless, we observed some relationship between the results of the docking to Ti IV, Ddl, and the antimicrobial activities of Ros. This finding allows for us to suggest the 1,2,4-triazole-5-thione core, with a 3-fluorophenyl moiety as a possible scaffold, for the further development of new antimicrobial agents with multiple mechanisms of action, which would be beneficial to the prevention of possible development of resistant forms. The docking scores of the investigated compounds with the selected enzymes are highlighted in the Table 4. 

**Figure 1:**
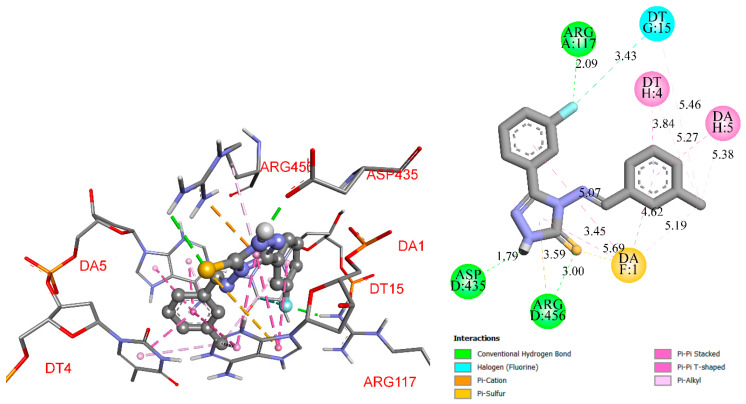
RO9-Topoisomerase IV interaction (three-dimensional and 2D scheme).

RO9, the most potent antibacterial compound from the set, exhibited binding abilities to both Ti IV and DR, which could be useful in reducing the risk of bacterial antibiotic resistance. The compound forms three hydrogen bonds with Arg117 (2.09 Å), Asp117 (2.09 Å), and Arg456 (3.00 Å). Fluorine bonds to the thymidine via the halogen bond. It also has a triazole core with the *m*-methylbenzylideneamino substituent and is held in place by hydrophobic interactions (Pi-cation, Pi-Sulfur, Pi-Pi Stacked, Pi-Pi T-shaped, Pi-Alkyl) (Figure 1).

**Figure 2:**
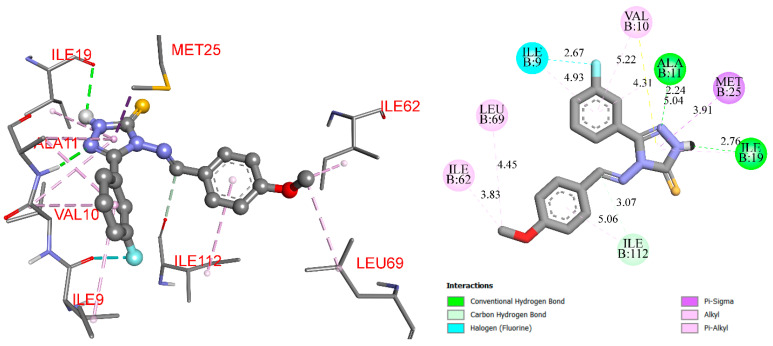
RO4-Dihydrofolate reductase interaction (3D and 2D scheme).

The RO4 compound possesses antifungal properties, which can be associated with the inhibition of dihydrofolate reductase. The activity is moderate, but the compound can serve as a basis for the development of new antifungal agents with different modes of action and as an alternative to 14α-lanosterol demethylase inhibitors, which are widely used but can lead to resistance in fungal infections. RO4 forms two hydrogen bonds with Ala11 (2.24 Å) and Ile19 (2.76 Å). The fluorine in the *meta* position interplays with the oxygen of Ile9 via the halogen bond. The 4-methoxy substituent of the phenyl ring stabilizes the position of the ligand inside the protein by Alkil and Pi-Alkyl’s hydrophobic interactions with the Leu69 and Ile62 (Figure 2). 

## 3. Materials and Methods

### 3.1. Chemistry

#### 3.1.1. General Comments

All the substances were purchased from Sigma-Aldrich (Munich, Germany) and used without further purification. The ^1^H and ^13^C NMR spectra were recorded on the Bruker AVANCE III 600 MHz (Bruker BioSpin GmbH, Rheinstetten, Germany) in DMSO-d_6_. Copies of the ^1^H NMR, ^13^C NMR spectra of the obtained compounds are available in the online Supplementary Materials as Figures S1–S20. IR spectra were recorded by a Nicolet 6700 spectrometer (Thermo Scientific, Philadelphia, PA, USA). The melting points were determined on the Stuart SMP50 melting point apparatus (Cole Parmer Ltd., Stone, UK) and were uncorrected. The purity of the compounds and the progress of the reaction were monitored by TLC (aluminum sheet 60 F254 plates; Merck Co., Kenilworth, NJ, USA). We used the solvent system CHCl_3_/EtOH (10:1, *v*/*v*). The elemental analyses were determined using a PerkinElmer 2400 series II CHNS/O analyzer (Waltham, MA, USA). Four of the obtained compounds had been described by other authors (RO1, RO4, RO7, and RO9), but we provide their physicochemical characteristics because they were not described for three compounds and were different for one compound.

#### 3.1.2. Synthesis of 4-Amino-5-(3-fluorophenyl)-2,4-dihydro-3H-1,2,4-triazole-3-thione

A 0.01 mole of 3-fluorobenzoic acid and 0.015 mole of solid potassium hydroxide were dissolved in 25 mL of anhydrous ethanol. The flask with the solution was placed on a magnetic stirrer in an ice bath and cooled to a temperature of 0–5 °C. A total of 1 mL of carbon disulfide was added dropwise. The mixture was mixed for 2 min. The precipitate was filtered off, washed with diethyl ether, and left to dry in the air. The potassium salt obtained was then subjected to a reaction with 80% hydrazine hydrate (10 mL) and heated with the substrates for 4 h under a reflux condenser. The solution was then cooled to room temperature, 50 mL of water was added, and the mixture was acidified with dilute hydrochloric acid. The precipitate was filtered off and, after drying in the air, crystallized from 96% ethanol. 4-Amino-5-(3-fluorophenyl)-2,4-dihydro-3*H*-1,2,4-triazole-3-thione was obtained with a yield of 80%. The physicochemical data of the compound were in agreement with the literature [49].

#### 3.1.3. Synthesis of Schiff Base Derivatives

A 0.01 mole of 4-amino-5-(3-fluorophenyl)-2,4-dihydro-3*H*-1,2,4-triazole-3-thione and 10 mL of anhydrous ethanol were placed in a round-bottom flask and heated to obtain a clear solution. A molar amount of the appropriate aldehyde was then added *. After that, the solutions were left at room temperature. The precipitates of compounds RO1, RO3, RO4, RO11, and RO12 were obtained immediately. The precipitates of compounds RO8 and RO10 were obtained after one hour. The precipitates of compounds RO6, RO9, and RO4 were obtained after 2 h. The precipitate of compound RO7 was obtained after 24 h. Despite many attempts, both at room temperature and at the boiling point (2, 4, 6, 8, 12 h), and the addition of hydrochloric acid as a reaction catalyst, two compounds RO2 and RO5 could not be obtained. The progress of the reaction was checked using thin-layer chromatography. After 24 h at room temperature, the resulting precipitate was filtered off and crystallized with 96% ethyl alcohol.

5-(3-fluorophenyl)-4-[(4-methylbenzylidene)amino]-2,4-dihydro-3*H*-1,2,4-triazole-3-thione (RO1) [50] CAS 1631040-03-3

Yield 81%, m.p. 227–230 °C. Spectral data were as follows: IR (cm^−1^) KBr: 3104 (NH), 2935 (CH aliph.), 1603(C=N), 1565 (CH arom.), 1358 (C=S). ^1^H NMR (DMSO-d_6_) δ (ppm): 2.41(s, 3H, CH_3_), 7.31–7.45 (m, 3H, ArH), 7.57–7.83 (m, 5H, ArH), 9.63 (s, 1H, =CH), 14.33 (s, 1H, NH). ^13^C NMR (DMSO-d_6_) δ (ppm): 21.76, 115.46 (d, *J* = 24.7 Hz), 118.02, 118.16, 124.85 (d, *J* = 2.9 Hz), 127.96, 128.02, 129.26, 129.64, 130.36, 131.47 (d, *J* = 8.6 Hz), 143.91, 147.78 (d, *J* = 2.9 Hz), 161.45, 163.05 (d, *J* = 5.3 Hz), 167.63. Elemental analysis for C_16_H_13_FN_4_S. Calculated: C 61.52; H 4.19; N 17.93. Found: C 61.30; H 4.10; N 17.80.

5-(3-fluorophenyl)-4-[(4-nitrobenzylidene)amino]-2,4-dihydro-3*H*-1,2,4-triazole-3-thione (RO3)

Yield 80%, m.p. 230–231 °C. Spectral data were as follows: IR (cm^−1^) KBr: 3114 (NH), 1593 (C=N), 1342 (NO_2_), 1559 (CH arom.), 1343 (C=S). ^1^H NMR (DMSO-d_6_) δ (ppm): 7.43–7.46 (m, 1H, ArH), 7.59–7.63 (m, 1H, ArH), 7.70 (d, *J* = 10.1 Hz, 1H, ArH), 7.74 (d, *J* = 10.8 Hz, 1H, ArH), 8.17 (d, *J* = 5.7 Hz, 2H, ArH), 8.40 (d, *J* = 5.9 Hz, 2H, ArH), 10.07 (s, 1H, =CH), 14.44 (s, 1H, NH). ^13^C NMR (DMSO-d_6_) δ (ppm): 115.70 (d, *J* = 24.3 Hz), 118.28 (d, *J* = 21.1 Hz), 124.86, 125.12 (d, *J* = 2.9 Hz), 127.74, 127.80, 130.06, 130.30, 131.53 (d, *J* = 8.4 Hz), 138.23, 148.22, 150.17, 161.48, 163.04 (d, *J* = 16.9 Hz), 164.03. Elemental analysis for C_15_H_10_FN_5_O_2_S. Calculated: C 52.47; H 2.94; N 20.40. Found: C 52.35; H 2.80; N 20.20.

5-(3-fluorophenyl)-4-[(4-methoxybenzylidene)amino]-2,4-dihydro-3*H*-1,2,4-triazole-3-thione (RO4) [51] CAS 2362542-60-5

Yield 79%, m.p. 215–216 °C. Spectral data were as follows: IR (cm^−1^) KBr: 3104 (NH), 2933(CH aliph.), 1599 (C=N), 1568 (CH arom.), 1361 (C=S), 1267 (C-O-C). ^1^H NMR (DMSO-d_6_) δ (ppm): 3.86 (s, 3H, OCH_3_), 7.13 (d, *J* = 8.6 Hz, 2H, ArH), 7.41 (t, *J* = 8.6 Hz, 1H, ArH), 7.56–7.62 (m, 1H, ArH), 7,70 (d, *J* = 8.6 Hz, 1H, ArH), 7.75 (d, *J* = 7.9 Hz, 1H, ArH), 7.87 (d, *J* = 8,6 Hz, 2H, ArH), 9.51 (s, 1H, =CH), 14.29 (s, 1H, NH). ^13^C NMR (DMSO-d_6_) δ (ppm): 56.07, 115.29, 115.47, 118.06 (d, *J* = 21.2 Hz), 124.73, 124.80, 128.04 (d, *J* = 8.7 Hz), 131.28, 131.47 (d, *J* = 8.2 Hz), 147.69, 161.45, 163.07, 163.61, 167.58. Elemental analysis for C_16_H_13_FN_4_OS. Calculated: C 58.52; H 4.00; N 17.06. Found: C 58.35; H 4.10; N 17.20.

5-(3-fluorofenylo)-4-[(4-propoxybenzylidene)amino]-2,4-dihydro-3*H*-1,2,4-triazole-3-thione (RO6)

Yield 53% (1.78 g), m.p. 189–192 °C. Spectral data were as follows: IR (cm^−1^) KBr: 3104 (NH), 2936 (CH aliph.), 1599 (C=N), 1568 (CH arom.), 1360 (C=S), 1262 (C-O-C). ^1^H NMR (DMSO-d_6_) δ (ppm): 1.00 (t, *J* = 7.4 Hz, 3H, CH_3_), 1.74–1.89 (m, 2H, CH_2_), 4.05 (t, *J* = 8.6 Hz, 2H, OCH_2_), 7.05 (d, *J* = 8.6 Hz, 1H, ArH), 7.12 (d, *J* = 8.8 Hz, 1H, ArH), 7.40–7.43 (m, 1H, ArH), 7.58–7.61 (m, 1H, ArH), 7.75 (d, *J* = 7.8 Hz, 1H, ArH), 7.80 (d, *J* = 8.6 Hz, 1H, ArH), 7.85 (d, *J* = 8.8 Hz, 2H, ArH), 9.49 (s, 1H, =CH), 14.30 (s, 1H, NH). ^13^C NMR (DMSO-d_6_) δ (ppm): 10.78, 22.36, 69.86, 115.30, 115.46, 115.70, 117.98, 118.12, 124.56, 124.79 (d, *J* = 3.1 Hz), 130.45, 131.30, 131.48 (d, *J* = 8.2 Hz), 160.94, 161.45, 161.60, 163.07, 167.66. Elemental analysis for C_18_H_17_FN_4_OS Calculated: C 60.70; H 4.80; N 15.70. Found: C 60.35; H 4.70; N 15.40.

4-[(4-fluorobenzylidene)amino]-5-(3-fluorophenyl)-2,4-dihydro-3*H*-1,2,4-triazole-3-thione (RO7) [37] CAS 1642872-25-0

Yield 81%, m.p. 217–218 °C. Spectral data were as follows: IR (cm^−1^) KBr: 3112 (NH), 1607 (C=N), 1585 (CH arom.), 1372 (C=S). ^1^H NMR (DMSO-d_6_) δ (ppm): 7.38–7.46 (m, 3H, ArH), 7.58–7.62 (m, 1H, ArH), 7.70 (d, *J* = 8.0 Hz, 1H, ArH), 7.74 (d, *J* = 8.1 Hz, 1H, ArH), 7.98–8.01 (m, 2H, ArH), 9.71 (s, 1H, =CH), 14.37 (s, 1H, NH). ^13^C NMR (DMSO-d_6_) δ (ppm): 115.50 (d, *J* = 24.3 Hz), 117.04 (d, *J* = 22,0 Hz), 118.15 (d, *J* = 20.8 Hz), 124.91 (d, *J* = 2.9 Hz), 127.89, 127.84, 131.50 (d, *J* = 8.6 Hz), 131.84 (d, *J* = 9.2 Hz), 147.8 (d, *J* = 2.9 Hz), 161.46, 163.05 (d, *J* = 7.5 Hz), 164.50, 166.33. Elemental analysis for C_15_H_10_F_2_N_4_S Calculated: C 56.95; H 3.19; N 17.71. Found: C 57.2; H 3.10; N 17.60.

4-[(4-butoxybenzylidene)amino]-5-(3-fluorophenyl)-2,4-dihydro-3*H*-1,2,4-triazole-3-thione (RO8)

Yield 56%, m.p. 183–185 °C. Spectral data were as follows: IR (cm^−1^) KBr: 3105 (NH), 2937 (CH aliph.), 1598 (C=N), 1569 (CH arom.), 1357 (C=S), 1255 (C-O-C). ^1^H NMR (DMSO-d_6_) δ (ppm): 0.95 (t, *J* = 7.4 Hz, 3H, CH_3_), 1.42–1.49 (m, 2H, CH_2_), 1.71–1.75 (m, 2H, CH_2_), 4.09 (t, *J* = 6.5 Hz, 2H, CH_2_), 7.12 (dd, *J* = 8.9, 2.2 Hz, 2H, ArH), 7.40–7.43 (m, 1H, ArH), 7.57–7.61 (m, 1H, ArH), 7.70 (d, *J* = 7.9 Hz, 1H, ArH), 7.75 (d, *J* = 7.6 Hz, 1H, ArH), 7.85 (dd, *J* = 8.9, 2.2 Hz, 2H, ArH), 9.49 (s, 1H, =CH), 14.29 (s, 1H, NH). ^13^C NMR (DMSO-d_6_) δ (ppm): 14.13, 19.14, 31.02, 68.11, 115.38 (d, *J* = 24.3 Hz), 115.70, 117.98, 118.05 (d, *J* = 21.1 Hz), 118.12, 124.79 (d, *J* = 2.8 Hz), 128.06 (d, *J* = 8.6 Hz), 131.29, 131.48 (d, *J* = 8.6 Hz), 147.68, 161.45, 163.08, 167.65. Elemental analysis for C_19_H_19_FN_4_OS. Calculated: C 61.60; H 5.17; N 15.12. Found: C 61.5; H 5.10; N 15.20.

5-(3-fluorophenyl)-4-[(3-methylbenzylidene)amino]-2,4-dihydro-3*H*-1,2,4-triazole-3-thione (RO9) [51] CAS 1631041-01-1

Yield 85%, m.p. 232–234 °C. Spectral data were as follows: IR (cm^−1^) KBr: 3105 (NH), 2938 (CH aliph.), 1603 (C=N), 1565 (CH arom.), 1358 (C=S). ^1^H NMR (DMSO-d_6_) δ (ppm): 2.41 (s, 3H, CH_3_), 7.40 (d, *J* = 7.7 Hz, 2H, ArH), 7.42–7.44 (m, 1H, ArH), 7.58–7.62 (m, 1H, ArH), 7.69–7.71 (m, 1H ArH), 7.75 (d, *J* = 9.1 Hz, 1H, ArH), 7.80 (d, *J* = 8.2 Hz, 2H, ArH), 9.62 (s, 1H, =CH), 14.33 (s, 1H, NH). ^13^C NMR (DMSO-d_6_) δ (ppm): 21.76, 115.38, 115.54, 118.10 (d, *J* = 20.8 Hz), 124.86 (d, *J* = 3.0 Hz), 127.97, 128.03, 129.27, 129.64, 130.37, 131.48 (d, *J* = 8.6 Hz), 143.91, 147.78 (d, *J* = 3.0 Hz), 161.45, 163.05 (d, *J* = 5.1 Hz), 167.66. Elemental analysis for C_16_H_13_FN_4_S Calculated: C 61.52; H 4.19; N 17.93 Found: C 61.35; H 4.10; N 17.80.

5-(3-fluorophenyl)-4-[(2-methylbenzylidene)amino]-2,4-dihydro-3*H*-1,2,4-triazole-3-thione (RO10)

Yield 85%, m.p. 223–224 °C. Spectral data were as follows: IR (cm^−1^) KBr: 3105 (NH), 2938 (CH aliph.), 1603 (C=N), 1565 (CH arom.), 1358 (C=S). ^1^H NMR (DMSO-d_6_) δ (ppm): 2.52 (s, 3H, CH_3_), 7.36–7.40 (m, 2H, ArH), 7.43 (td, *J* = 8.6, 2.7 Hz, 1H, ArH), 7.51 (t, *J* = 7.4 Hz, 1H, ArH), 7.59–7.63 (m, 1H, ArH), 7.71 (dt, *J* = 10.1, 2.1 Hz, 1H, ArH), 7.75 (d, *J* = 8.2 Hz, 1H, ArH), 7.92 (d, *J* = 7.7 Hz, 1H, ArH), 10.03 (s, 1H, =CH), 14.37 (s, 1H, NH). ^13^C NMR (DMSO-d_6_) δ (ppm): 19.47, 115.63 (d, *J* = 24.3 Hz), 118.13 (d, *J* = 20.9 Hz), 125.01 (d, *J* = 2.9 Hz), 127.10, 127.29, 127.98 (d, *J* = 9.1 Hz), 130.62, 131.46 (d, *J* = 8.6 Hz), 131.75, 133.06, 140.09, 148.01 (d, *J* = 2.9 Hz), 161.44, 162.98 (d, *J* = 25.2 Hz), 165.97. Elemental analysis for C_16_H_13_FN_4_S Calculated: C 61.52; H 4.19; N 17.93Found: C 61.35; H 4.10; N 17.40.

5-(3-fluorophenyl)-4-[(3-methoxybenzylidene)amino]-2,4-dihydro-3*H*-1,2,4-triazole-3-thione (RO11)

Yield 90%, m.p. 203–204 °C. Spectral data were as follows: IR (cm^−1^) KBr: 3102 (NH), 2935 (CH aliph.), 1606 (C=N), 1577 (CH arom.), 1261 (C-O-C), 1362 (C=S). ^1^H NMR (DMSO-d_6_) δ (ppm): 3.83 (s, 3H, OCH_3_), 7.22–7.24 (m, 1H, ArH), 7.42–7.51 (m, 4H, ArH), 7.60–7.62 (m, 1H, ArH), 7.71–7.75 (m, 2H, ArH), 9.73 (s, 1H, =CH), 14.35 (s, 1H, NH). ^13^C NMR (DMSO-d_6_) δ (ppm): 55.81, 113.17, 115.56 (d, *J* = 24.3 Hz), 118.15 (d, *J* = 21.9 Hz), 119.55, 122.06, 122.08, 124.95, 127.89, 130.95, 131.48 (d, *J* = 8.6 Hz), 133.75, 160.16, 161.45, 162.99, 166.84. Elemental analysis for C_16_H_13_FN_4_OS. Calculated: C 58.52; H 4.00; N 17.06. Found: C 58.37; H 4.10; N 17.10.

5-(3-fluorophenyl)-4-[(2-methoxybenzylidene)amino]-2,4-dihydro-3*H*-1,2,4-triazole-3-thione (RO12)

Yield 62%, m.p. 140–143 °C. Spectral data were as follows: IR (cm^−1^) KBr: 3106 (NH), 2935(CH aliph.), 1594(C=N), 1572(CH arom.), 1351(C=S), 1254(C-O-C). ^1^H NMR (DMSO-d_6_) δ (ppm): 3.91 (s, 3H, OCH_3_), 7.08–7.12 (m, 1H, ArH), 7.20–7.24 (m, 1H, ArH), 7.37–7.43 (m, 1H, ArH), 7.56–7.64 (m, 2H, ArH), 7.65–7.75 (m, 2H, ArH), 7.91–7.95 (m, 1H, ArH), 10.04 (s, 1H, =CH), 14.29 (s, 1H, NH). ^13^C NMR (DMSO-d_6_) δ (ppm): 56.52, 112.94, 115.63 (d, *J* = 24.7 Hz), 118.03 (d, *J* = 21.4 Hz), 120.44, 121.51, 124.99 (d, *J* = 2.9 Hz), 127.12, 128.08 (d, *J* = 8.7 Hz), 131.39 (d, *J* = 8.3 Hz), 135.27, 147.98, 159.88, 161.42, 162.28, 162.96 (d, *J* = 24.2 Hz). Elemental analysis for C_16_H_13_FN_4_OS. Calculated: C 58.52; H 4.00; N 17.06. Found: C 58.41; H 4.01; N 17.20.

### 3.2. Microbiology

#### 3.2.1. General Comments

A total of 50 mg of the Schiff bases was dissolved in 1 mL dimethylsulphoxide (DMSO) for the preparation of stock solutions. Before detection of the antimicrobial activity of the newly synthesized compounds, the basic concentration of 1000 µg/mL with Mueller–Hinton broth medium without glucose (for bacteria), or supplemented with 2% glucose (for fungi), was prepared as a solvent of each compound. Then, a series of two-fold dilutions of the tested compounds in the concentration range from 0.49 to 1000 μg/mL was made in culture broth medium before the determination of antimicrobial activity.

The bacterial and fungal suspensions were prepared in sterile saline 0.85% NaCl with an optical density of McFarland Standard of 0.5 (approximately 150 × 10^6^ CFU (Colony Forming Units)/mL).

The reference antibiotics used during our studies for the selected species of reference bacteria were ciprofloxacin and cefuroxime (Merck KGaA, Darmstadt, Germany). Nystatin (Merck KGaA, Darmstadt, Germany), a known antifungal antibiotic, was used as a positive control for *Candida* spp. 

All reference antimicrobials against bacteria or fungi were tested in a final concentration range from 0.0049 to 10 µg/mL. The tested Schiff bases were two-fold diluted in the concentration range from 0.49 to 1000 μg/mL in culture broth medium for bacteria or fungi. The medium with DMSO at the final concentrations and without the tested Schiff bases served as a control—no microbial growth inhibition was observed. Three types of control were also carried out simultaneously: (1) serial dilution of the tested Schiff bases in broth medium (without bacteria or fungi) in the same concentration range, (2) a growth control for each strain in broth medium and agar medium for bacteria or fungi, and (3) a negative control of sterile broth or agar medium.

The antimicrobial activity of all the tested Schiff bases was determined based on the minimal inhibitory concentration (MIC) and the minimal bactericidal concentration (MBC) or the minimal fungicidal concentration (MFC). Minimal inhibitory concentrations (MICs) are defined as the lowest concentrations of an antimicrobial that will inhibit the visible growth of a microorganism (bacteria or fungi) after overnight incubation, and minimal bactericidal concentrations (MBCs) or minimal fungicidal concentrations (MFC) as the lowest concentrations of an antimicrobial that will prevent the growth of an organism (bacteria or fungi, respectively) after their subculture on antimicrobial-free media and incubation.

#### 3.2.2. Bacterial and Fungal Strains

Fresh overnight cultures of various aerobically grown reference strains of bacteria and yeasts from the American Type Culture Collection (ATCC) were used in all experiments. The panel of the tested microorganisms included Gram-positive bacteria: *Staphylococcus aureus* ATCC 6538, *Staphylococcus epidermidis* ATCC 12228, *Micrococcus luteus* ATCC 10240, and *Bacillus subtilis* ATCC 6633 and Gram-negative bacteria: *Escherichia coli* ATCC 25922, *Pseudomonas aeruginosa* ATCC 27853, and *Klebsiella pneumoniae* ATCC 13883. For the determination of antifungal activity, various reference strains of *Candida* spp. were used: *C. albicans* ATCC 10231, *C. glabrata* ATCC 15126, *C. glabrata* ATCC 90030, *C. krusei* ATCC 14243, *C. auris* CDC B11903, *C. lusitaniae* ATCC 3449, and *C. tropicalis* ATCC 1369. 

#### 3.2.3. Antimicrobial Activity Determination

##### Antibacterial Activity Determination

The minimal inhibitory concentration (MIC) was determined with the microdilution technique using 96-well polystyrene microplates (Medlab, Poland), as indicated by the European Committee on Antimicrobial Susceptibility Testing (EUCAST) guidelines [52]. In this process, stock solutions of each tested Schiff base dissolved in dimethylsulphoxide (DMSO, Stanlab, Poland) were used at a concentration of 50 mg/mL. Before in vitro antibacterial activity detection, the basic concentration of 1000 µg/mL of each compound was prepared with Mueller–Hinton broth medium (MHB, BioMaxima, Lublin, Poland). The experiment was prepared using Mueller–Hinton broth medium in 96-well microplates. A series of two-fold dilutions of the tested compounds in the concentration range from 0.49 to 1000 μg/mL was made in culture broth medium. Then, 2 μL of bacterial inoculum with a density of 0.5 McFarland standard was added to each well. Each well was filled with a total volume of 100 μL. Then, the microtiter plates were incubated for 18 ± 2 h at 35 °C. After the incubation, the results were spectrophotometrically determined in Biotek ELx800 (Biokom, Janki, Poland) at 600 nm (OD_600_). The lowest concentration of the tested compounds with the absence of bacterial growth was determined as an MIC. Three replicates for each strain and compound were performed.

##### Antifungal Activity Determination

To determine the antifungal activity of the newly synthesized Schiff bases against the reference strains of *Candida* spp., the broth microdilution method was used according to the EUCAST [52] and Clinical and Laboratory Standards Institute (CLSI) guidelines [53] with some modifications to the medium. Before in vitro antifungal activity detection, the basic concentration of 1000 µg/mL of each compound was prepared with Mueller–Hinton broth (BioMaxima, Lublin, Poland) supplemented with 2% glucose (MHB+2% glucose). Then, the MIC of the tested compounds was examined using their two-fold dilutions in Mueller–Hinton broth (BioMaxima, Lublin, Poland) with 2% glucose (MHB+2% glucose) prepared in 96-well polystyrene plates. The final concentrations of the Schiff bases ranged from 0.002 to 1000 µg/mL. All the reference strains of *Candida* spp. used were first subcultured to the Mueller–Hinton agar medium (BioMaxima, Lublin, Poland) with 2% glucose (MHA +2% glucose) at 37 °C for 24 h. Next, 2 µL of appropriate fungal suspensions was added to each well containing 100 µL of MHB + 2% glucose and the various concentrations of the tested Schiff bases. After incubation (37 °C, 24 h), the MIC was spectrophotometrically assessed, with OD_600_ as the lowest concentration of the samples inducing total fungal growth inhibition. Moreover, the MFC was observed to be the lowest concentration of the compounds required to kill a particular fungal species. 

##### Determination of the Minimum Bactericidal (MBC) or Fungicidal (MFC) Concentration

MBCs or MFCs were determined using the broth dilution from the MIC tests in subcultures in Mueller–Hinton Agar (BioMaxima, Lublin, Poland) medium for bacteria or Mueller–Hinton Agar medium supplemented with 2% glucose (MHA +2% glucose) plate for fungi. After incubation, 5 μL of the broth media cultured with bacteria or fungi were removed from each well of the plates used in the MIC experiments. Next, they were culturd onto the surface of the MHA medium (BioMaxima, Lublin, Poland) plate for bacteria or MHA +2% glucose plate for yeasts. The inoculated plates were incubated in the conditions mentioned above. After incubation, a visual reading of MBC or MFC values was made. The MBC or MFC was observed as the lowest concentration of the tested compounds with the absence of any visible bacterial (MBC) or fungal (MFC) growth on the MHA (for bacteria) or MHA +2% glucose (for fungi) plates. Three replicates for each strain and compound were performed.

All the experiments were repeated three times and representative data were presented. In this study, the MFC/MIC ratios were also calculated in order to determine the bactericidal and fungicidal (MBC/MIC and MFC/MIC ≤ 4) or bacteriostatic and fungistatic (MBC/MIC and MFC/MIC > 4) effect of the examined Schiff bases [54,55]. 

### 3.3. Cell Viability Assay

#### 3.3.1. General Comments

Compounds RO1-RO12 were tested on metastatic breast cancer (MDA-MB-231), prostate cancer (PC3), and human immortal keratinocyte (HaCaT) cell lines. All the cell lines were purchased from the American Type Culture Collection (ATCC, Rockville, MD, USA) and cultured in strictly defined conditions according to the recommended ATCC protocol. The PC3 cells were cultured in RPMI 1640 (Roswell Park Memorial Institute, Biowest SAS, Nuaillé, France), while the MDA-MB-231 and HaCaT cells were maintained in DMEM (Dulbecco’s Modified Eagle’s Medium, Biowest SAS, Nuaillé, France) supplemented with 1% streptomycin and penicillin and in 10% heat-inactivated fetal bovine serum (FBS, Gibco BRL San Francisco, CA, USA) with 1% streptomycin and penicillin. The cells were incubated in a 37 °C/5% CO_2_ humidified incubator. They were cultured until 80–90% confluence was achieved. Then, they were harvested by treatment with 0.25% trypsin-0.02% EDTA (Gibco BRL, San Francisco, CA, USA) and used in the experiments. Untreated cells were used as a control.

#### 3.3.2. MTT Assay

The cell viability was assessed by the determination of MTT salt [3-(4,5-dimethylthiazol-2-yl)-2,5-diphenyltetrazoliumbromide] conversion by mitochondrial dehydrogenase. After 24 h of incubation, the culture medium was replaced with a fresh medium. Then, all compounds RO1-RO12 and reference cytostatics doxorubicin and cisplatin were added at various concentrations (range from 10 to 100 µM) on 96-well plates (0.5 × 10^4^ cells per well) with seeded MDA-MB-231, PC3, HaCaT cells and cultured to adhere for 48 h at 37 °C in a CO_2_ incubator. Then, the medium was aspirated and formed formazan crystals were solubilized by adding isopropanol-DMSO mixture (1:1 vol). The intensity of the dissolved crystals was measured using a UVM 340 reader (ASYS Hitech GmbH, Eugendorf, Austria) at a wavelength of 570 nm. Cell viability was estimated by the percentage of MTT reduction in cells to which the tested compounds were added, compared to the control sample, where only the medium was added. The experiments were repeated three times. The IC_50_ was calculated using GraphPad Prism 9.0.0 (GraphPad Software).

### 3.4. Docking

**Figure 3:**
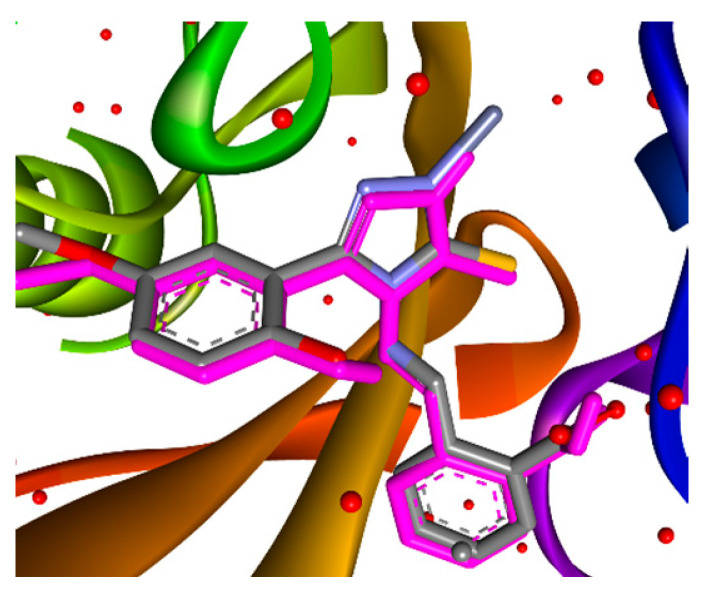
Real and predicted (pink-colored) position of the PJQ inside MBL (PBD 6YRP).

Docking simulations were performed to explore the possible mode of action and the possible ways of improving the antibacterial and antifungal activity of the synthesized compounds. Antibacterial Topoisomerase IV (PDB code 3LTN–Ti IV), D-Alanyl-D-Alanine Ligase (PDB code 1IOV–Ddl), Dihydrofolate reductase of *Candida albicans* (PDB code 1M7A–DR), and Metallo-β-lactamase (PDB code 6YRP–MBL) were chosen as target enzymes, owing to the presence of reported inhibitors for these pharmacological targets with related structures [56,57,58,59]. The X-ray spectra were downloaded from the Protein DataBank. (https://www.rcsb.org/09.012023, accessed on 27 January 2023). All ligands were subjected to the energy minimization procedure performed using the Austin Model 1 (AM1) [60] semi-empirical quantum technique in the HyperChem 7.5 software package. The optimized molecules were converted by OpenBabel into the suitable PDBQT format for further docking studies with AutoDock (v4.2.6). The enzyme structures were prepared for the simulation with the appropriate procedure: all water molecules and additional ligands were removed from the structures, polar hydrogens were added, non-polar hydrogens were merged, and Kollman charges were added and spread over the residues. A Lamarckian Genetic Algorithm (LGA) was employed for docking simulations [61]. Docking parameters were set as the default, apart from the number of GA runs (increased from 10 to 50) and the population size (increased from 150 and 300). The receptor grid files for each enzyme were generated with a grid box size of 55 × 55 × 55 Å. With the aim of evaluating the selected docking techniques and parameters, a redocking of the co-crystallized inhibitors was performed. The RMSD parameters ≤ 2 Å [62] were the indicators of the ability of the AutoDock v4.2.6 tools to generate appropriate docking poses of the tested compounds inside the selected enzymes. The MBL enzyme possesses Zn ions inside its structure, and the reference ligand forms donor–acceptor bonds. The newest AutoDock Vina 1.2.3 with the special AutoDock4 force-field [63] was used for the docking studies, which now enables docking on zinc metalloenzymes. This updated version employs the placement of fictional points (TZ) to complement the Zn coordination spheres, thereby enabling the anchoring of the ligands’ 1,2,4-triazole-3-thione core [64]. The docking techniques used allow for the reproduction of the position of the reference ligand inside the MBL enzyme with high accuracy (Figure 3). Estimations of the compound’s potent inhibitory activity were considered in comparison with binding energies and inhibition constants Ki of the native ligands from selected enzymes. Only binding energies were used to estimate the MBL inhibition activity. Discovery Studio Visualizer v. 21.1 was used as molecular visualization software for displaying and analyzing the predicted protein–ligand complexes. 

## 4. Conclusions

In conclusion, in the pool of tested compounds, molecules with selective antifungal activity and those with activity directed towards opportunistic pathogens, including staphylococci, can be distinguished. The greatest advantage of the newly synthesized bioactive molecules is the selectivity of their antimicrobial action. Many research groups are focusing on new antimicrobials and therapeutic approaches, which will offer another tool in the fight against opportunistic pathogens, including new compounds and targets in microbial cells or new antimicrobial strategies under clinical development. The highest therapeutic potential was determined for molecules with activity against *Candida* spp., including *C. albicans*. In this context, the selectivity of antifungal agents is important, preventing an additional imbalance in the patient’s bacterial flora. The RO1, RO4, and RO12 molecules seem to be promising structures in relation to these therapeutic problems, due to their high and selective activity against *C. albicans*. All compounds showed very low toxicity to human cells. This high selectivity, combined with low toxicity, makes these molecules potential “lead-hit” structures for the further development of new antifungal agents. Compounds with antifungal activity and no antibacterial and cytotoxic activity could be helpful in the treatment of opportunistic yeast infections in immunocompromised patients, e.g., after oncological treatment or those with AIDS.

This paper focuses on promising new chemical compounds with activity against opportunistic organisms, which are innovative in comparison with traditional antimicrobials. The docking simulations demonstrated a possible multitarget mode of the antibacterial activity of the synthesized compounds. The antifungal properties may be connected to the inhibition of dihydrofolate reductase.

## Figures and Tables

**Table 1:** Activity of the newly synthesized Schiff bases against reference strains of bacteria expressed as the values of the minimal inhibitory concentration (MIC; µg/mL) and the minimal bactericidal concentration (MBC; µg/mL).

Compounds	*Staphylococcus aureus* ATCC 6538		*Staphylococcus epidermidis* ATCC 12228		*Micrococcus luteus* ATCC 10240		*Bacillus* *subtilis* ATCC 6633		*Escherichia coli* ATCC 25922		*Klebsiella pneumoniae* ATCC 13883		*Pseudomonas aeruginosa* ATCC 27853	
	**MIC**	**MBC**	**MIC**	**MBC**	**MIC**	**MBC**	**MIC**	**MBC**	**MIC**	**MBC**	**MIC**	**MBC**	**MIC**	**MBC**
RO1	>1000	nd *	>1000	nd *	>1000	nd *	>1000	nd *	>1000	nd *	>1000	nd *	>1000	nd *
RO3	>1000	nd *	>1000	nd *	>1000	nd *	>1000	nd *	>1000	nd *	>1000	nd *	>1000	nd *
RO4	500	>1000	500	>1000	125	>1000	1000	nd *	1000	nd *	1000	nd *	>1000	>1000
RO6	1000	nd *	1000	nd *	250	>1000	1000	nd *	1000	nd *	1000	nd *	1000	nd *
RO7	500	>1000	500	>1000	250	>1000	>1000	nd *	>1000	nd *	>1000	nd *	>1000	nd *
RO8	250	>1000	250	>1000	125	>1000	1000	nd *	1000	nd *	>1000	nd *	>1000	nd *
RO9	125	>1000	250	>1000	125	>1000	500	>1000	>1000	nd *	>1000	nd *	>1000	nd *
RO10	125	>1000	1000	nd *	>1000	nd *	>1000	nd *	>1000	nd *	>1000	nd *	>1000	nd *
RO11	>1000	nd *	>1000	nd *	>1000	nd *	>1000	nd *	>1000	nd *	>1000	nd *	>1000	nd *
RO12	>1000	nd *	>1000	nd *	>1000	nd *	>1000	nd *	>1000	nd *	>1000	nd *	>1000	nd *

* nd, not determined.

**Table 2:** Activity of the newly synthesized Schiff bases against reference strains of yeasts expressed as the values of the minimal inhibitory concentration (MIC; µg/mL) or minimal fungicidal concentration (MFC; µg/mL).

Compounds	*Candida* *albicans* ATCC *10231*		*Candida* *glabrata* ATCC *90030*		*Candida* *glabrata* ATCC 15126		*Candida* *krusei* ATCC 14243		*Candida* *auris* CDC B11903		*Candida* *lusitaniae* ATCC 3449		*Candida* *tropicalis* ATCC 1369	
	**MIC**	**MFC**	**MIC**	**MFC**	**MIC**	**MFC**	**MIC**	**MFC**	**MIC**	**MFC**	**MIC**	**MFC**	**MIC**	**MFC**
RO1	250	>1000	>1000	nd *	>1000	nd *	>1000	nd *	>1000	nd *	>1000	nd *	>1000	nd *
RO3	500	nd *	>1000	nd *	1000	nd *	1000	nd *	1000	nd *	1000	nd *	500	nd *
RO4	62.5	>1000	>1000	nd *	1000	nd *	500	>1000	1000	nd *	1000	nd *	1000	nd *
RO6	1000	nd *	>1000	nd *	1000	nd *	500	>1000	500	>1000	>1000	nd *	>1000	nd *
RO7	>1000	nd *	>1000	nd *	>1000	nd *	>1000	nd *	>1000	nd *	>1000	nd *	>1000	nd *
RO8	500	>1000	>1000	nd *	500	>1000	>1000	nd *	>1000	nd *	>1000	nd *	>1000	nd *
RO9	1000	>1000	>1000	nd *	>1000	nd *	>1000	nd *	>1000	nd *	>1000	nd *	>1000	nd *
RO10	500	>1000	500	>1000	500	>1000	>1000	nd *	1000	nd *	>1000	nd *	500	>1000
RO11	500	1000	500	>1000	>1000	nd *	1000	nd *	1000	nd *	>1000	nd *	1000	nd *
RO12	125	>1000	500	>1000	>1000	nd *	1000	nd *	500	>1000	500	>1000	500	>1000

* nd, not determined.

**Table 3:** Cytotoxic activity (IC_50_ µM/µg/mL) of the studied compounds estimated by the MTT assay ^a^.

Compound	MDA-MB-231 ^d^		PC3 ^e^		HaCaT ^f^
	IC_50_^b^	SI ^c^	IC_50_	SI	IC_50_
RO1	>100 µM/ 31.21 µg/mL	1	>100 µM/ 31.21 µg/mL	1	>100 µM/ 31.21 µg/mL
RO3	>100 µM/ 34.33 µg/mL	1	>100 µM/ 34.33 µg/mL	1	>100 µM/ 34.33 µg/mL
RO4	>100 µM/ 38.84 µg/mL	1	>100 µM/ 38.84 µg/mL	1	>100 µM/ 38.84 µg/mL
RO6	>100 µM/ 35.64 µg/mL	1	>100 µM/ 35.64 µg/mL	1	>100 µM/ 35.64 µg/mL
RO7	>100 µM/ 31.63 µg/mL	1	>100 µM/ 31.63 µg/mL	1	>100 µM/ 31.63 µg/mL
RO8	>100 µM/ 37.04 µg/mL	1	>100 µM/ 37.04 µg/mL	1	>100 µM/ 37.04 µg/mL
RO9	>100 µM/ 31.21 µg/mL	1	>100 µM/ 31.21 µg/mL	1	>100 µM/ 31.21 µg/mL
RO10	>100 µM/ 31.21 µg/mL	1	>100 µM/ 31.21 µg/mL	1	>100 µM/ 31.21 µg/mL
RO11	>100 µM/ 38.84 µg/mL	1	>100 µM/ 38.84 µg/mL	1	>100 µM/ 38.84 µg/mL
RO12	>100 µM/ 38.84 µg/mL	0.98	>100 µM/ 38.84 µg/mL	0.98	98.08 µM ± 0.5/38.09 µg/mL
Doxorubicin ^g^	0.83 µM ± 0.03	0.36	0.3 µM ± 0.1	1	0.3 µM ± 0.1
Cisplatin ^h^	3.95 µM ± 1.1	1.59	13.2 µM ± 2.1	0.47	6.3 µM ± 0.7

^a^ Data are expressed as mean SD. ^b^ IC_50_ (µM)—the concentration of the compound that corresponds to 50% growth inhibition of the cell line (compared to the control) after culturing the cells for 72 h with the individual compound. ^c^ The SI (Selectivity Index) was calculated using the formula: SI = IC_50_ for the normal cell line/IC_50_ the cancer cell line. ^d^ Human breast adenocarcinoma (MDA-MB-231), ^e^ Human metastatic prostate cancer (PC3), ^f^ Human immortal keratinocyte cell line from adult human skin (HaCaT). ^g,h^ Reference compounds.

**Table 4:** AutoDock docking scores (binding energies and inhibition constants) for the synthesized compounds.

Compounds	Ti IV PDB 3LTN		Ddl PDB 1IOV		DR PDB 1M7A		MBL PBD 6YRP
	Binding Energy	Inhibition Constants, µM	Binding Energy	Inhibition Constants, µM	Binding Energy	Inhibition Constants, µM	Binding Energy
RO1	−8.32	0.792	−8.41	0.688	−8.82	0.339	−7.23
RO3	−7.45	3.49	−6.77	10.81	−6.15	30.81	−7.29
RO4	−8.45	0.639	−8.24	0.907	−9.35	0.139	−6.89
RO6	−8.03	1.30	−7.99	1.39	−8.02	1.31	−7.18
RO7	−8.24	0.905	−8.13	1.09	−7.52	3.05	−7.19
RO8	−8.68	0.436	−8.71	0.412	−7.75	2.09	−7.07
RO9	−9.25	0.165	−9.16	0.192	−7.74	2.11	−7.31
RO10	−8.99	0.258	−8.47	0.621	−7.71	2.23	−7.38
RO11	−8.46	0.629	−8.80	0.357	−8.97	0.266	−7.33
RO12	−8.62	0.483	−8.38	0.721	−9.00	0.254	−7.12
PDQ ^1^	−12.31	0.00094	-	-	-	-	-
POB ^2^	-	-	−9.79	0.067	-	-	-
MQU ^3^	-	-	-	-	9.82	0.063	-
PJQ ^4^							−7.18

^1,2,3,4^—PDQ, POB, MQU, PJQ–PDB codes of appropriate co-crystallized inhibitors of the tested enzymes.

## Data Availability

The details of the data supporting the report results in this research were included in the paper and Supplementary Materials.

## Supplementary Materials

The following supporting information can be downloaded at: https://www.mdpi.com/article/10.3390/molecules28062718/s1: ^1^H NMR (Figures S1–S10) and ^13^C NMR (Figures S11–S20) spectra.

## Sample Availability

Samples of the compounds are available from the authors upon request.
