# Supplementary material for: Synthesis and Biological Evaluation of New Schiff Bases Derived from 4-Amino-5-(3-fluorophenyl)-1,2,4-triazole-3-thione
Source: Molecules. 2023 Mar 17;28(6):2718. doi: 10.3390/molecules28062718 (PMC10057893; doi:10.3390/molecules28062718)
Supplement: Supplementary file 1 [file molecules-28-02718-s001.zip › molecules-2230263-supplementary.pdf]

Supplementary Information

Sara Janowska [1], Dmytro Khylyuk [1], Michał Janowski [1], Urszula Kosikowska [2], Paulina Strzyga-Łach [3], Marta Struga [3] and Monika Wujec [1,*]

[1] Department of Organic Chemistry, Faculty of Pharmacy, Medical University, 4a Chodzki Str., 20-093 Lublin, Poland
[2] Department of Pharmaceutical Microbiology, Faculty of Pharmacy, Medical University, 1 Chodzki Str., 20-093 Lublin, Poland
[3] Chair and Department of Biochemistry, Faculty of Medicine, Banacha Str. 1, 02-097 Warsaw, Poland
***** Correspondence: monika.wujec@umlub.pl

Table of contents:

1. ^{1}H NMR spectra ……………………………..……………………………………2
2. ^{13}C NMR spectra ……..………………………………………………………….....12

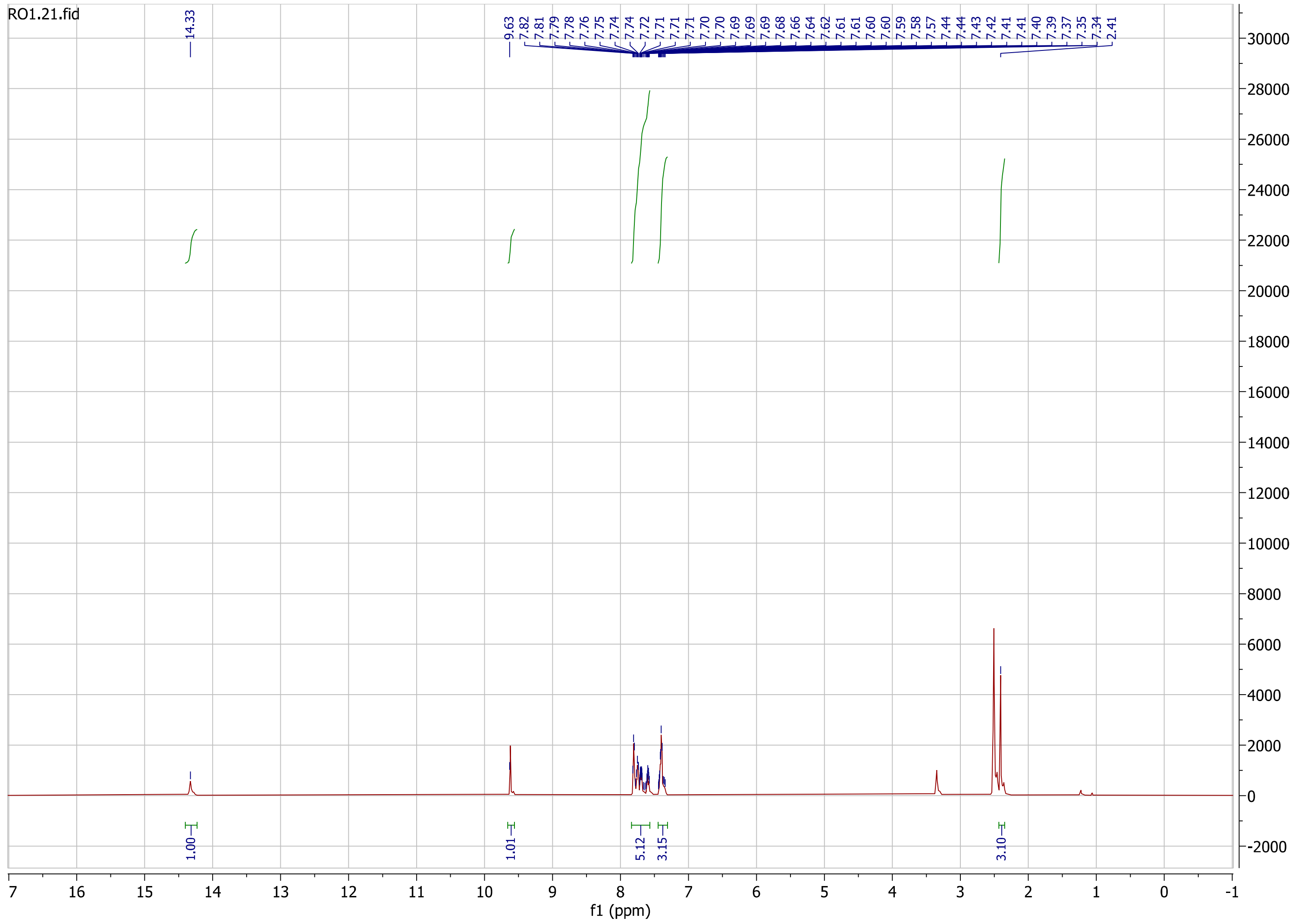

Figure S1. The ^{1}H NMR of compound RO1.

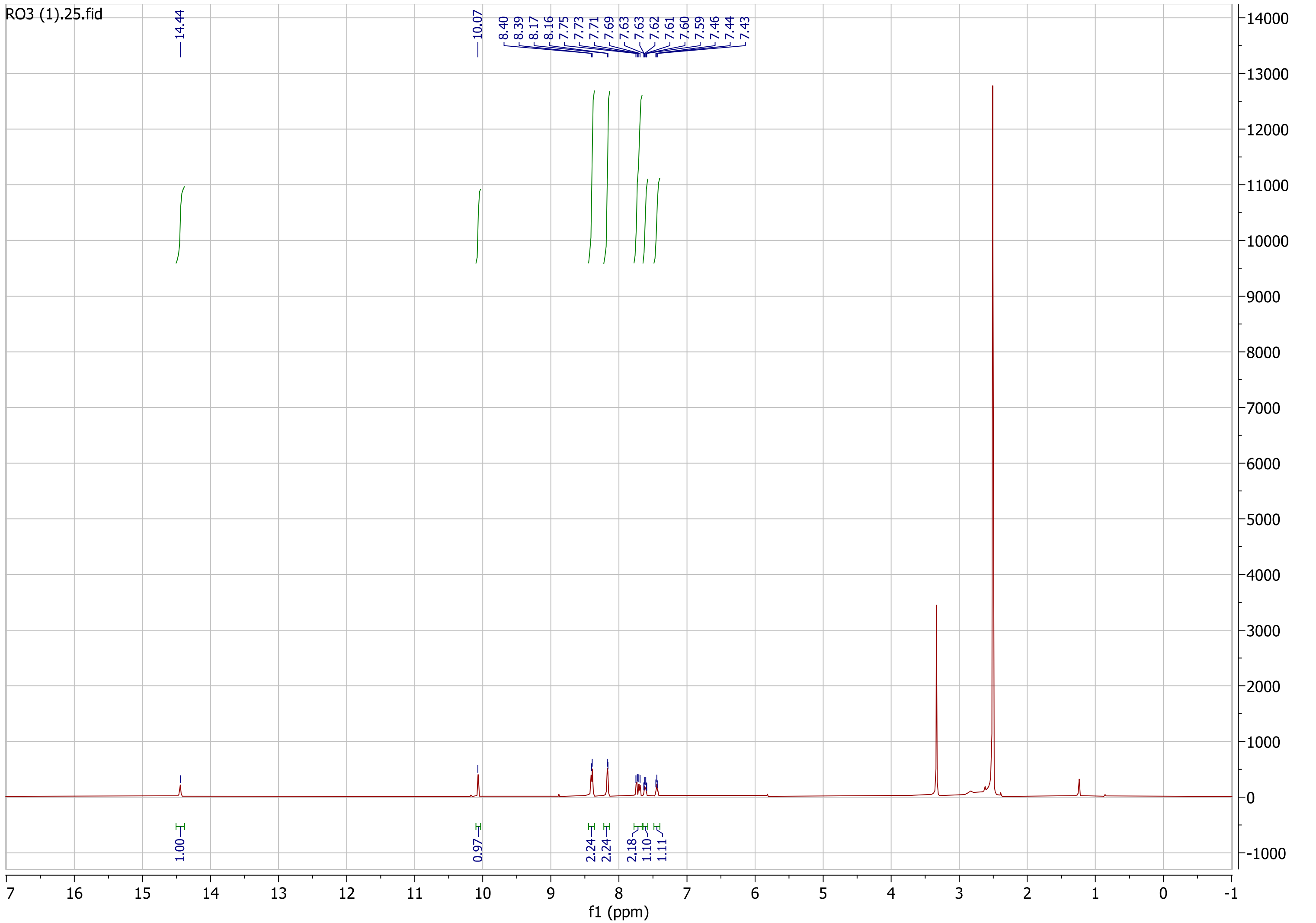

Figure S2. The ^{1}H NMR of compound RO3.

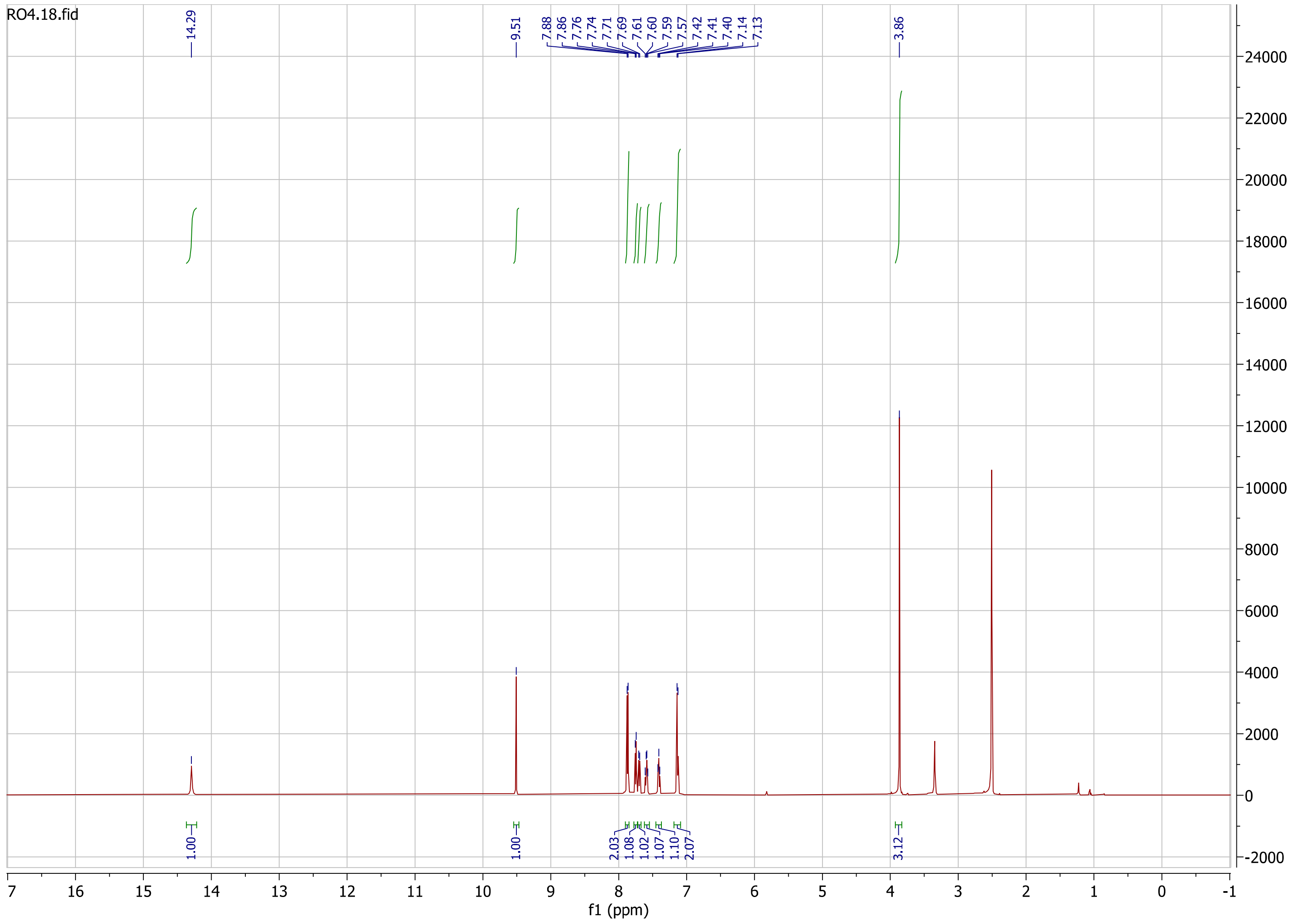

Figure S3. The ^{1}H NMR of compound RO4.

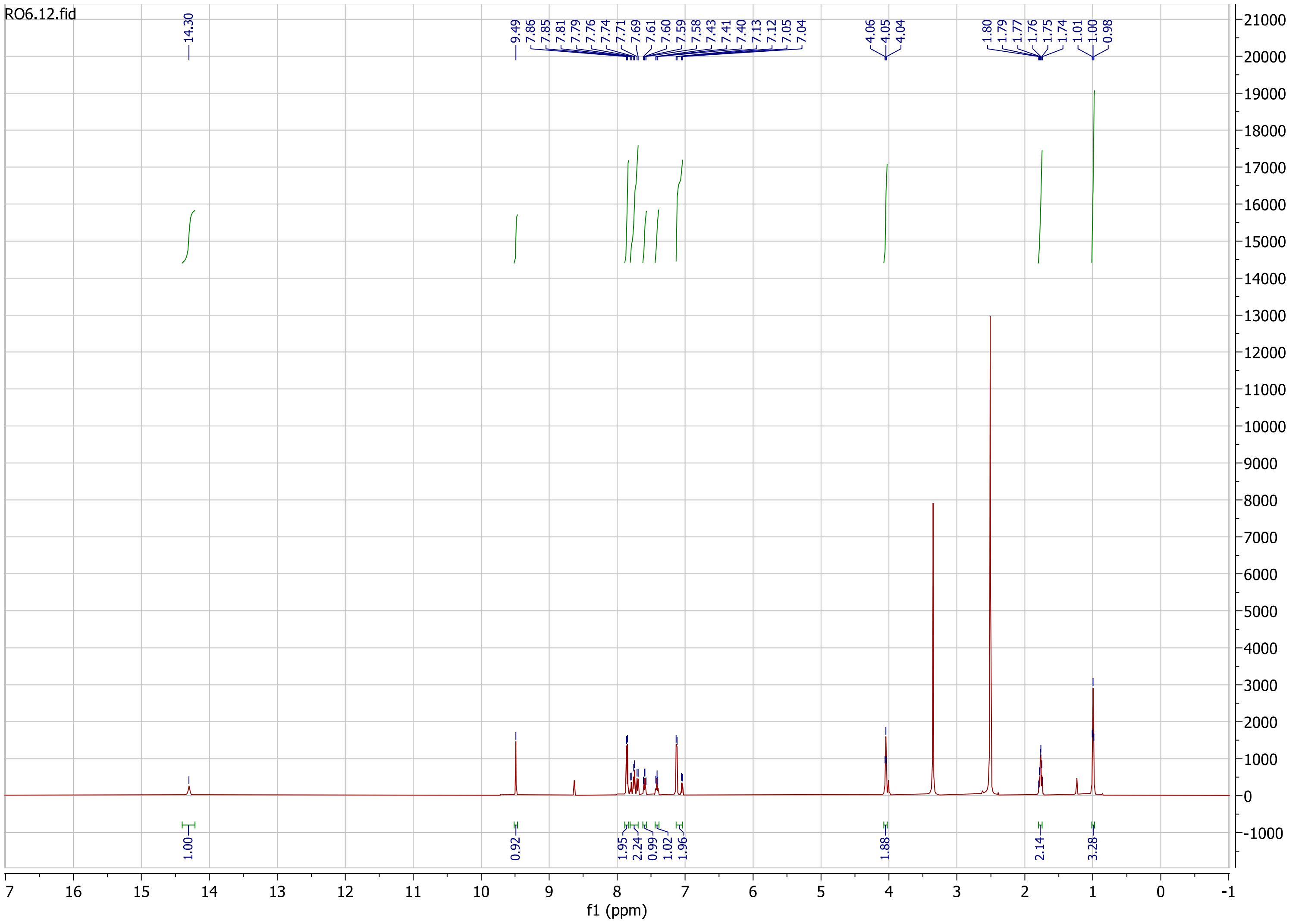

Figure S4. The ^{1}H NMR of compound RO6.

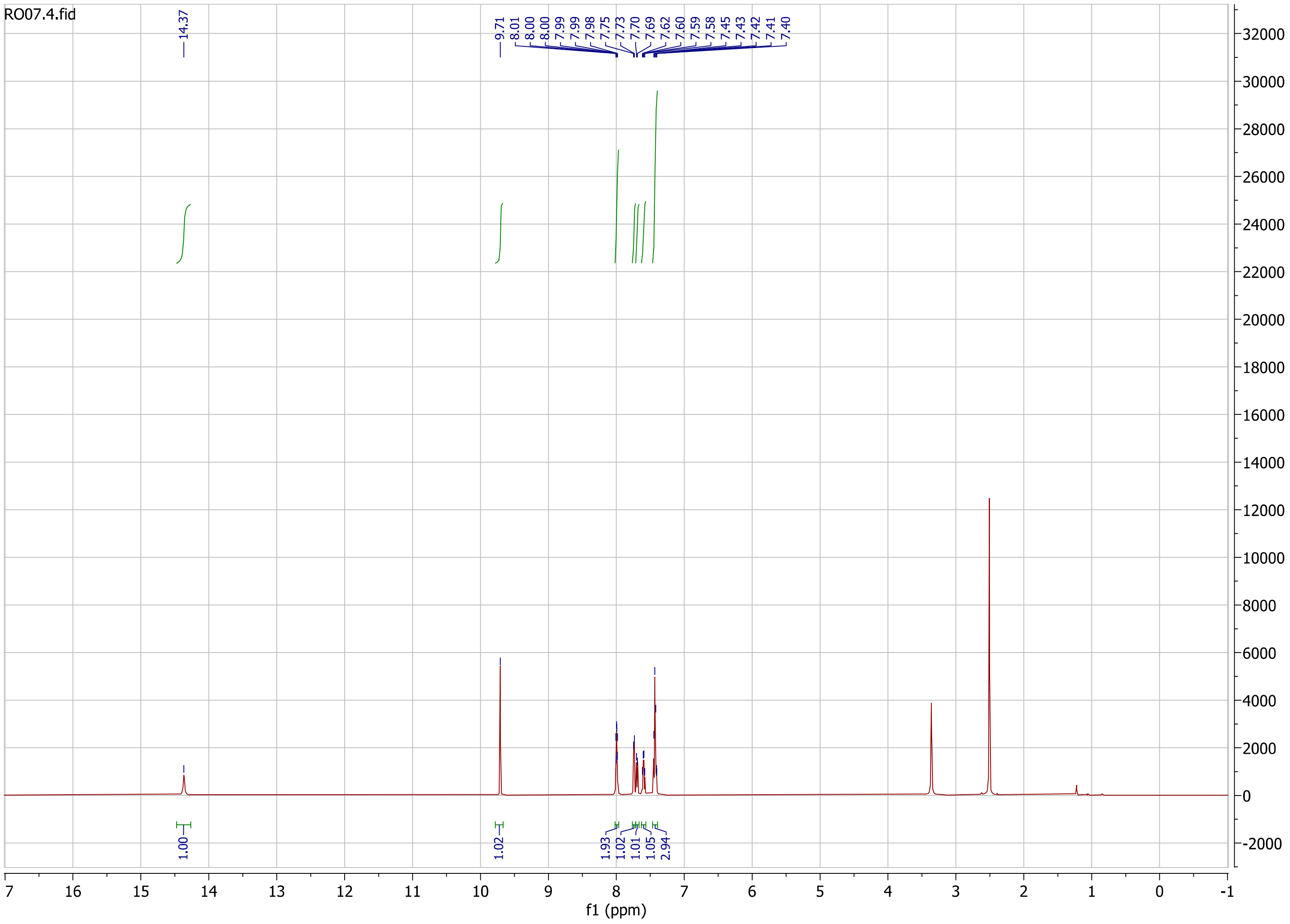

Figure S5. The ^{1}H NMR of compound RO7.

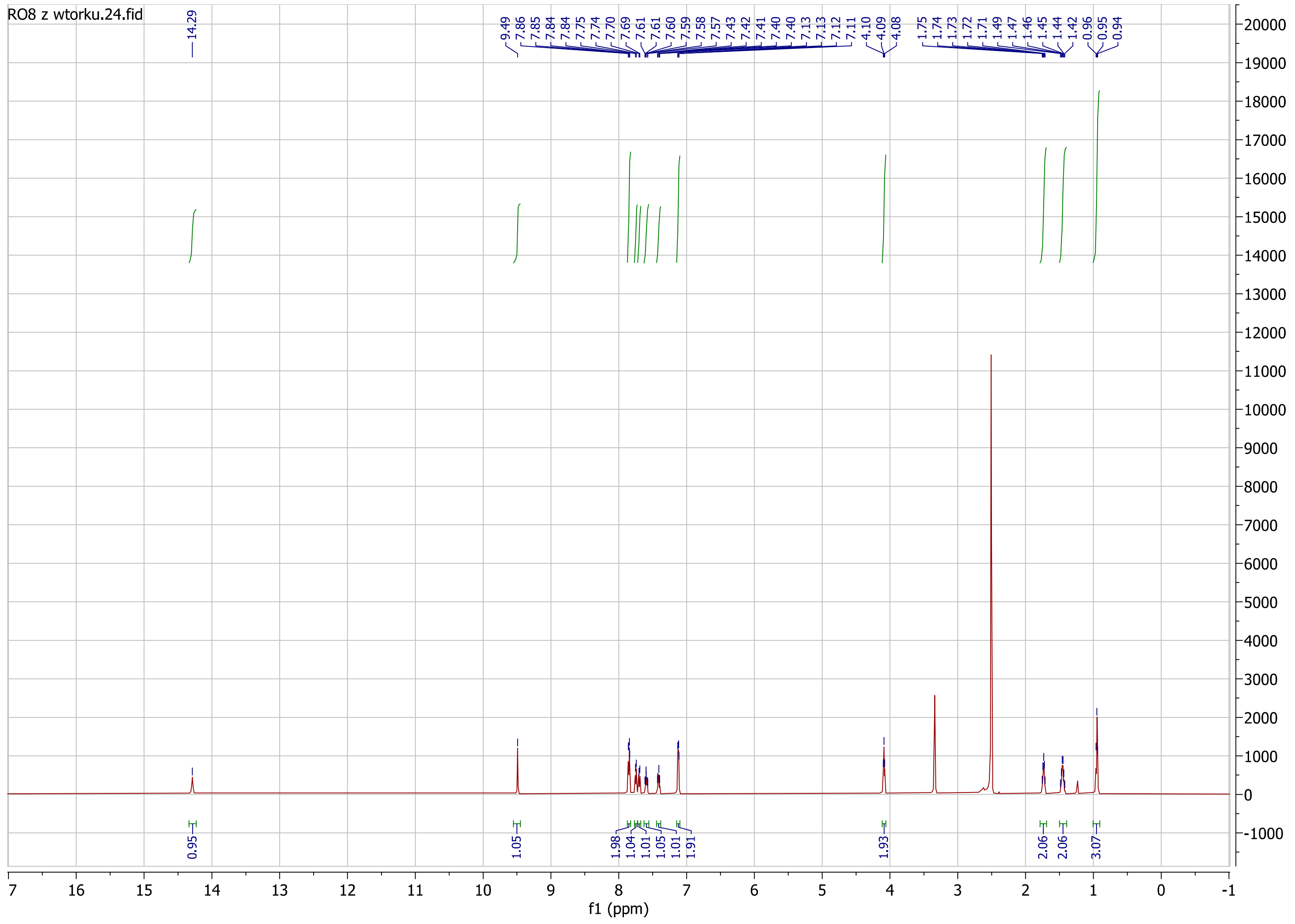

Figure S6. The ^1H NMR of compound RO8.

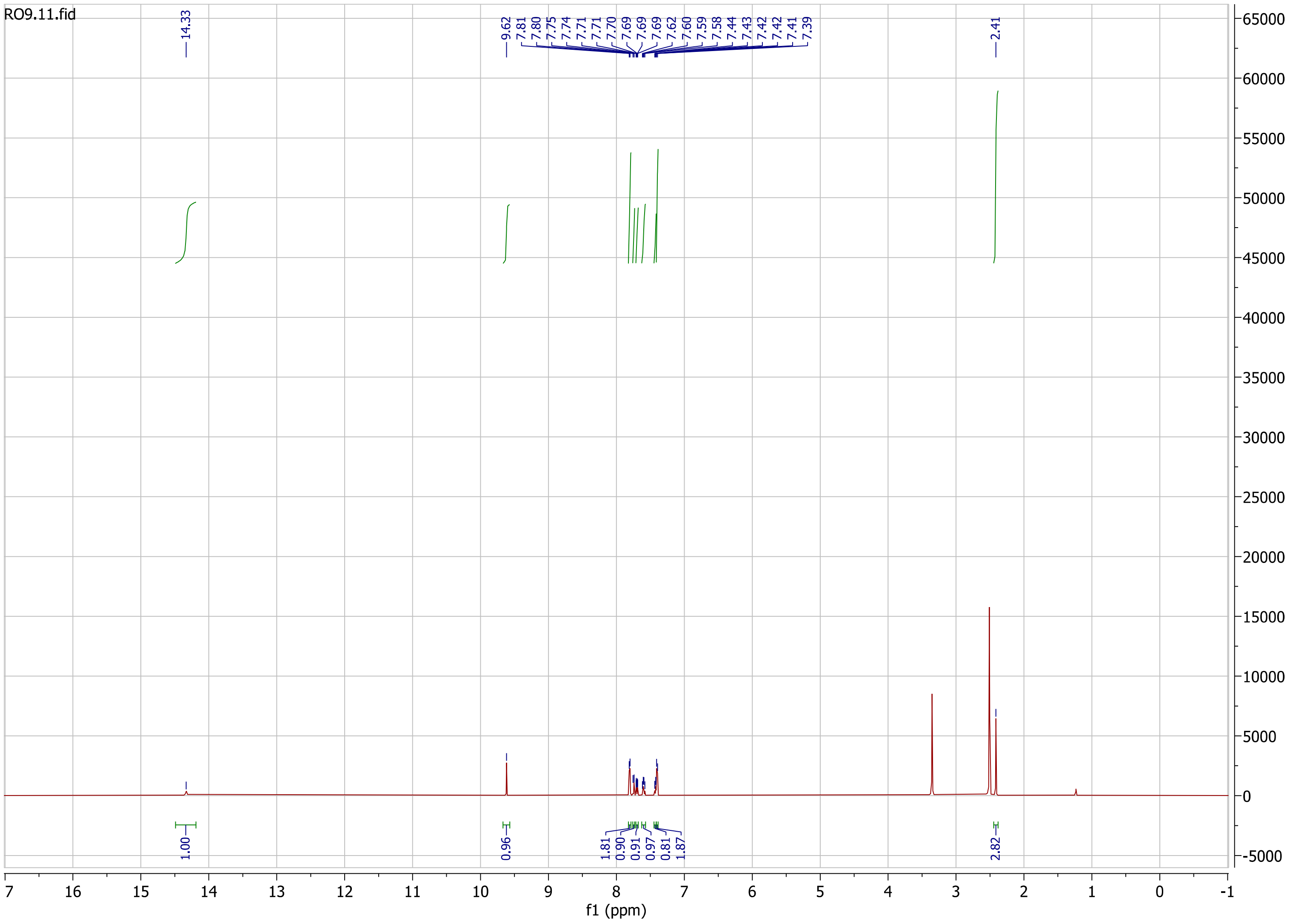

Figure S7. The ^{1}H NMR of compound RO9.

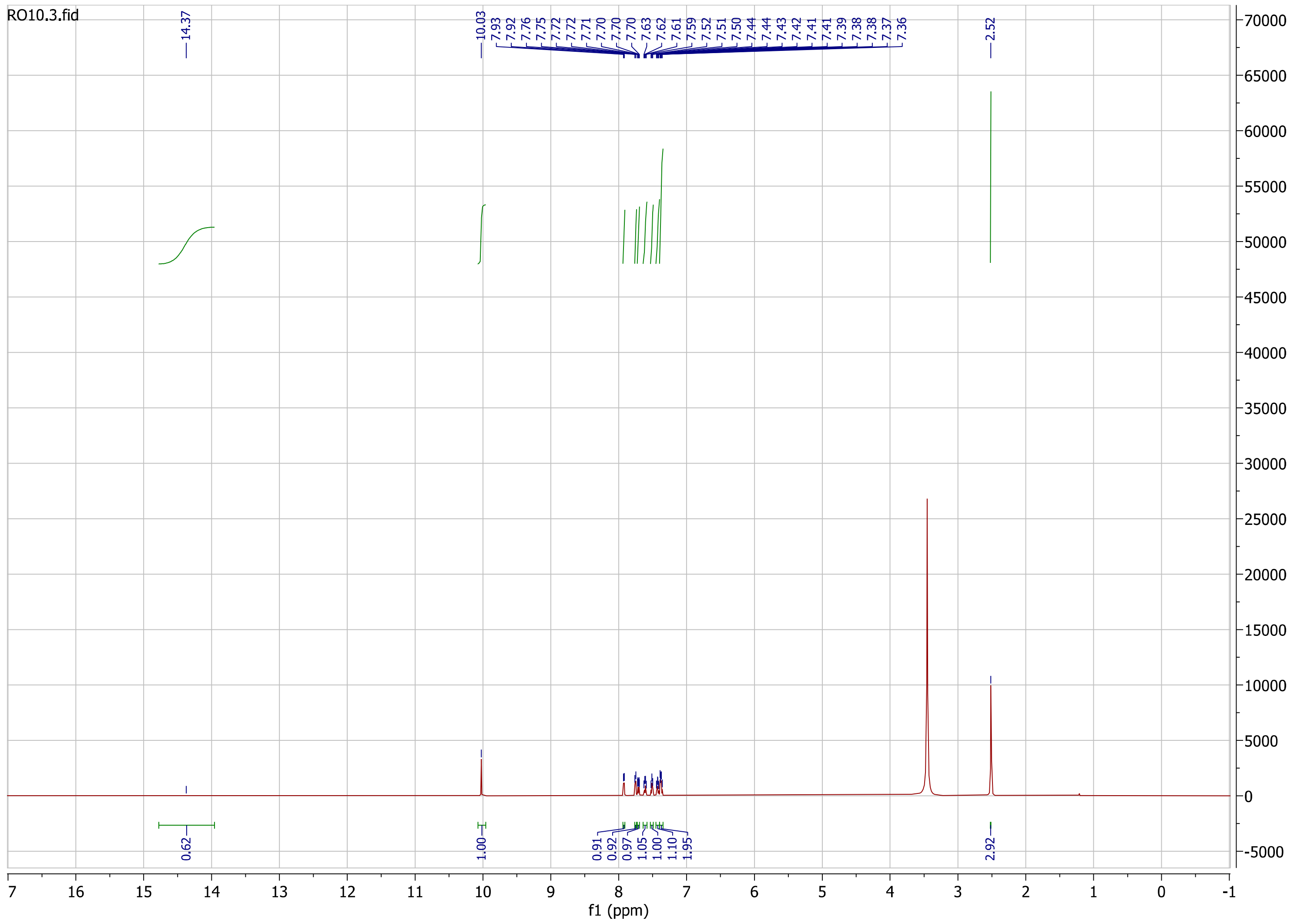

Figure S8. The ^{1}H NMR of compound RO10.

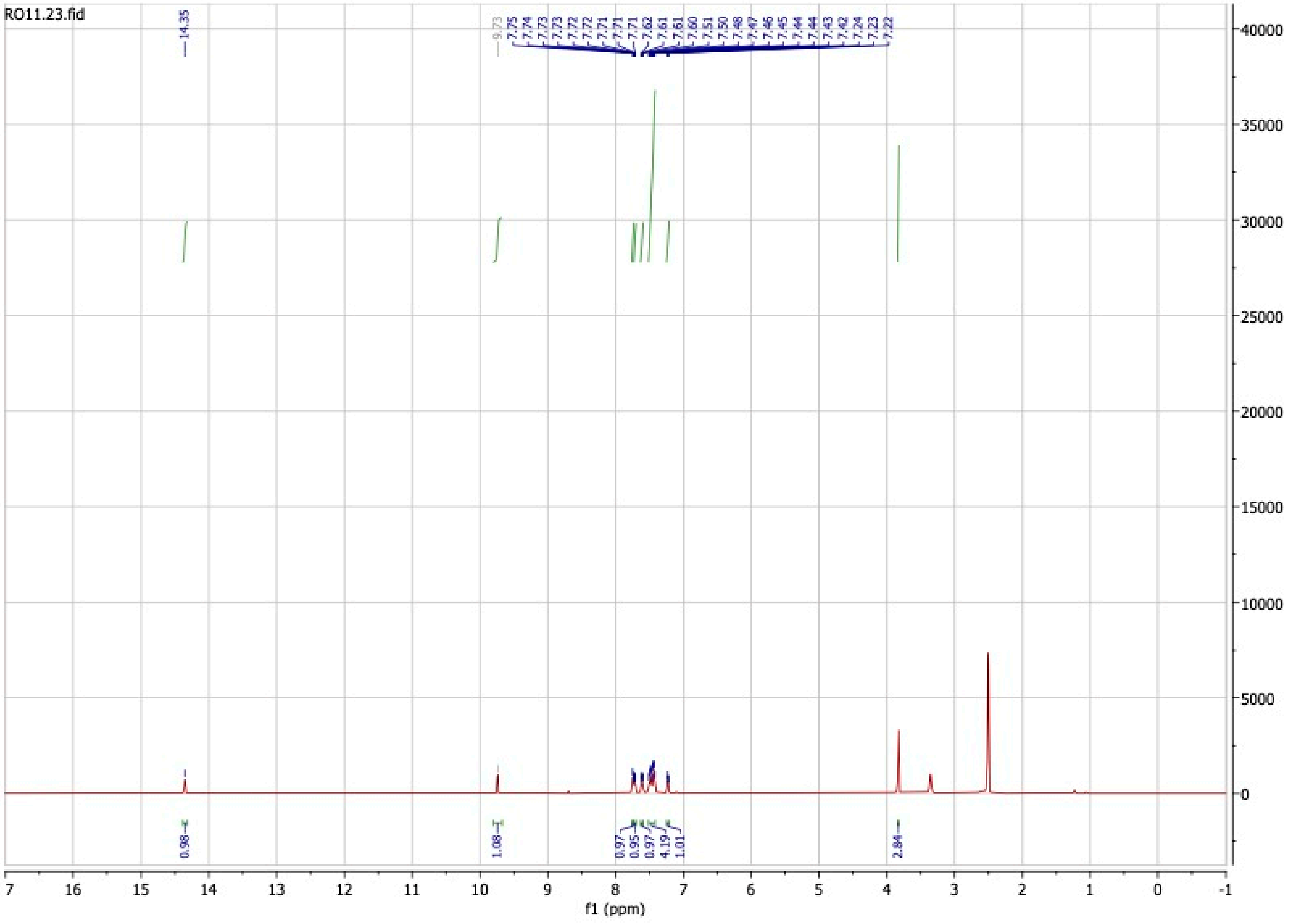

Figure S9. The ^{1}H NMR of compound RO11.

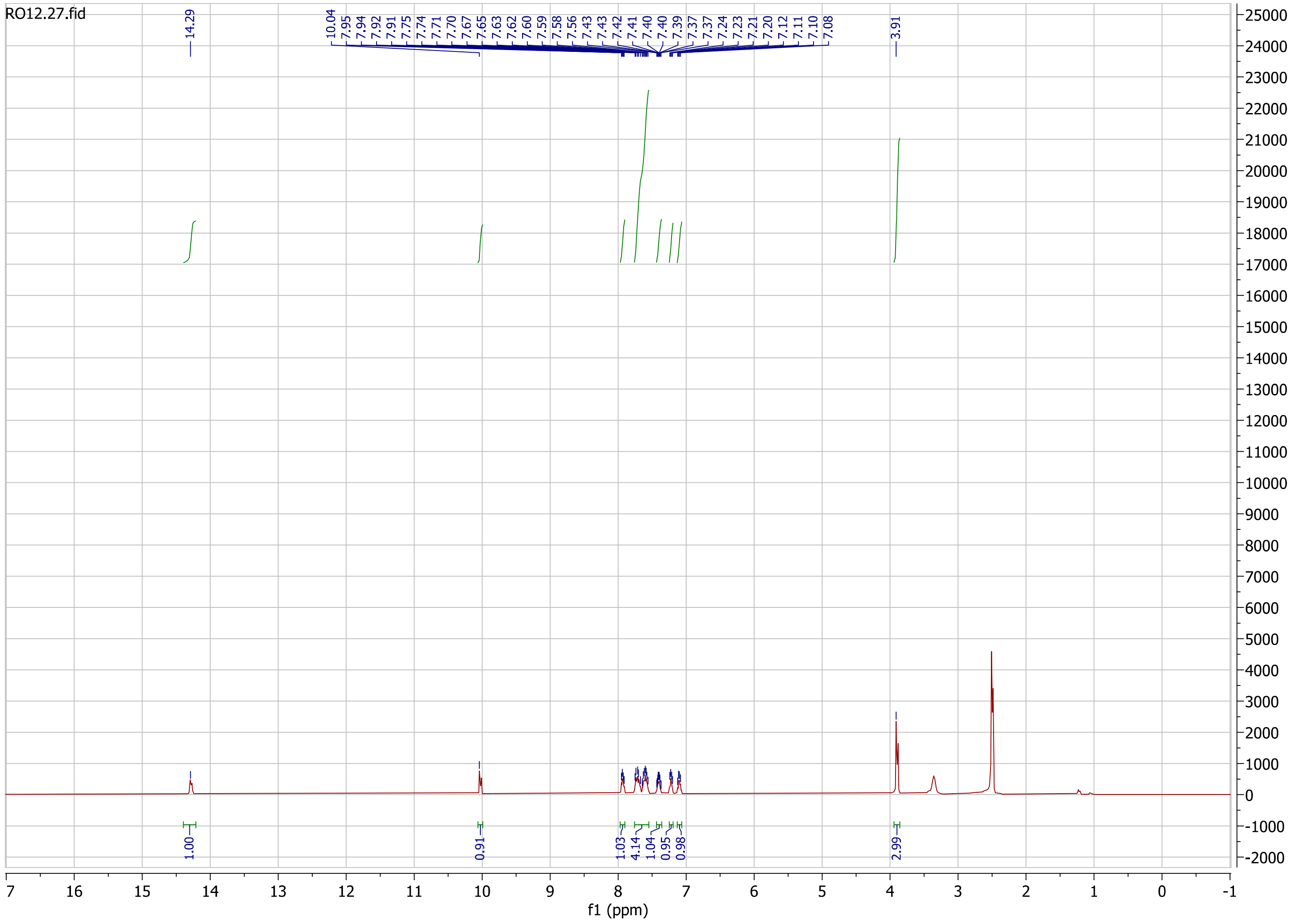

Figure S10. The ¹H NMR of compound RO12.

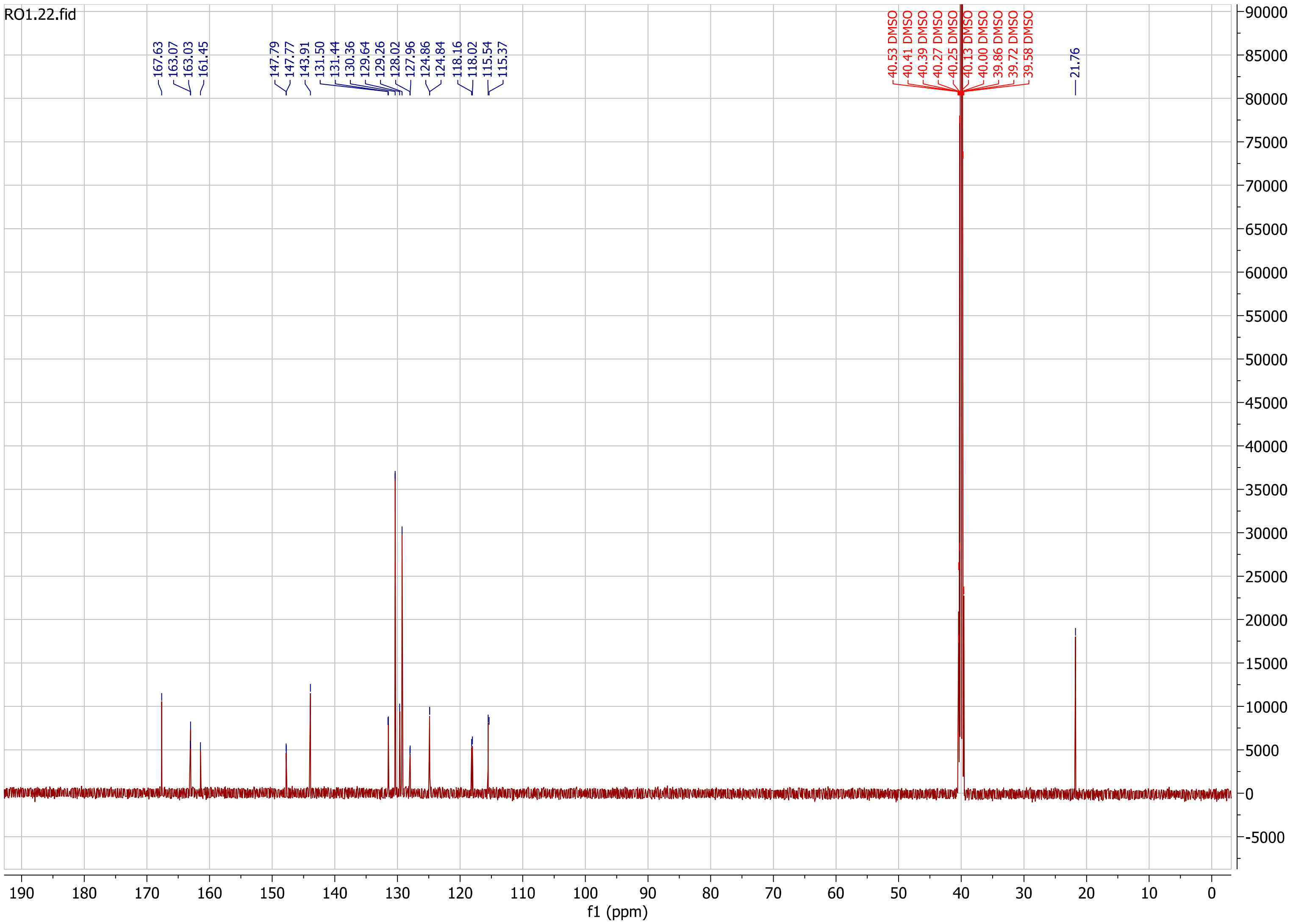

Figure S11. The ^{13}C NMR of compound RO1.

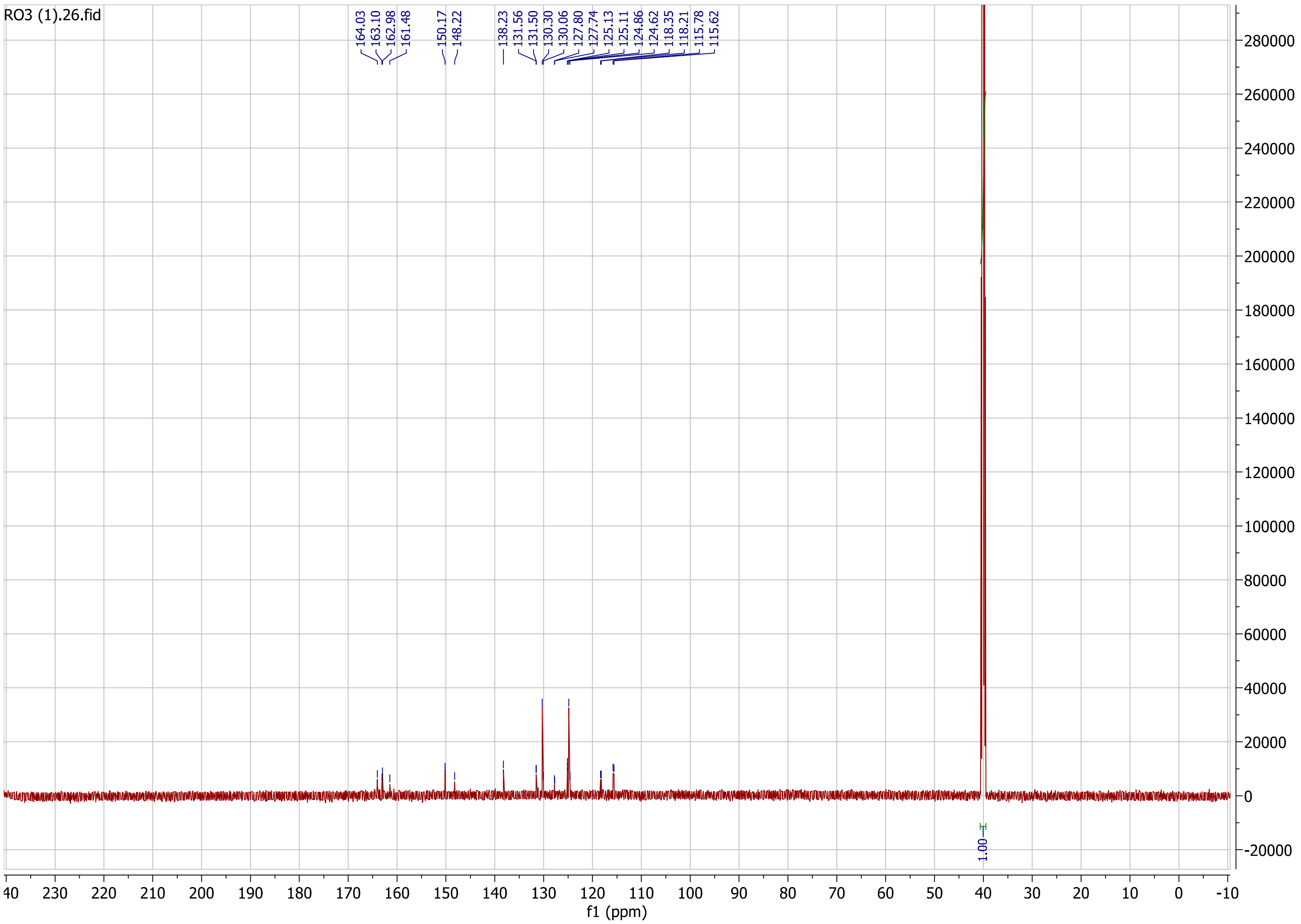

Figure S12. The ^{13}C NMR of compound RO3.

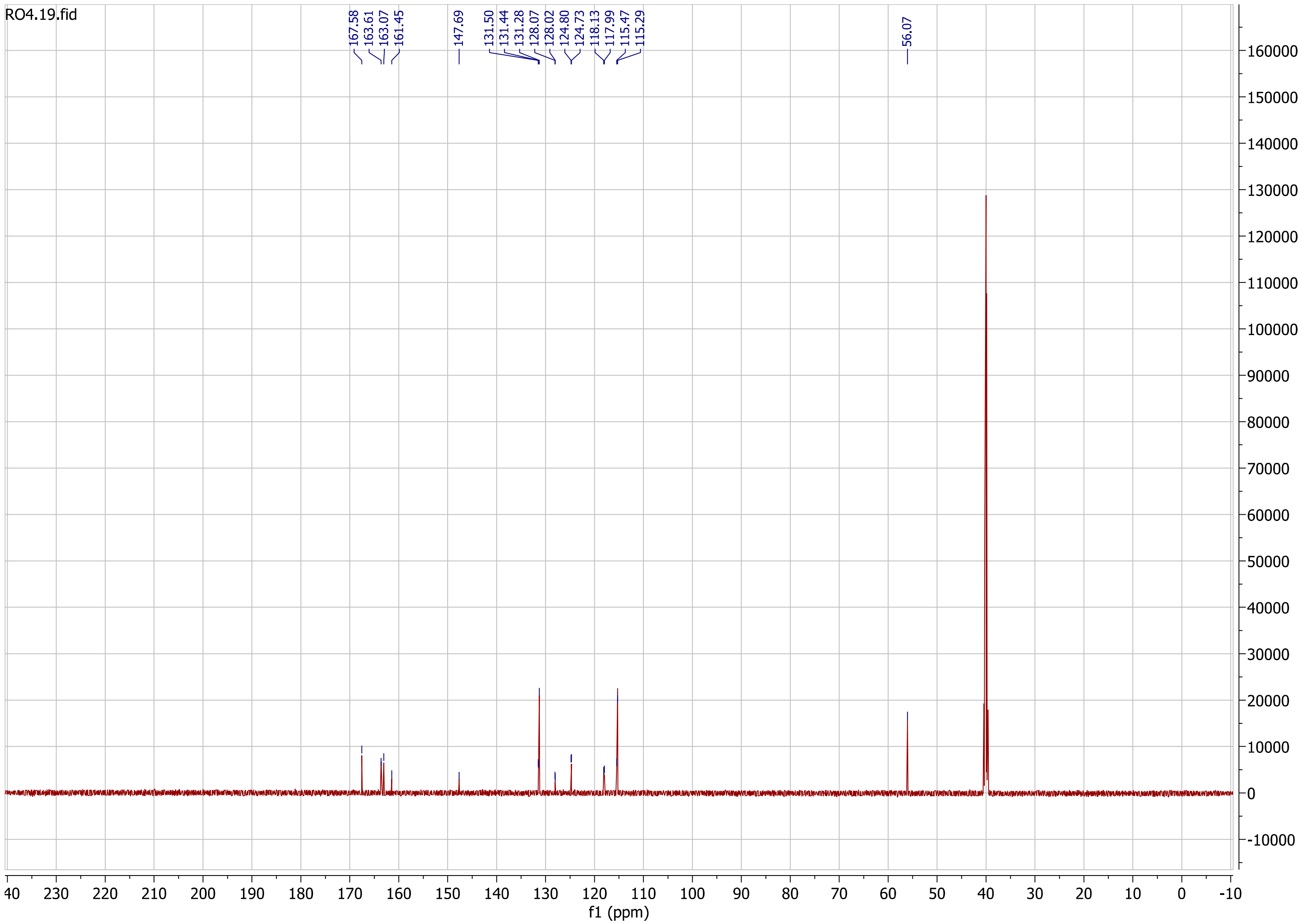

Figure S13. The ^{13}C NMR of compound RO4.

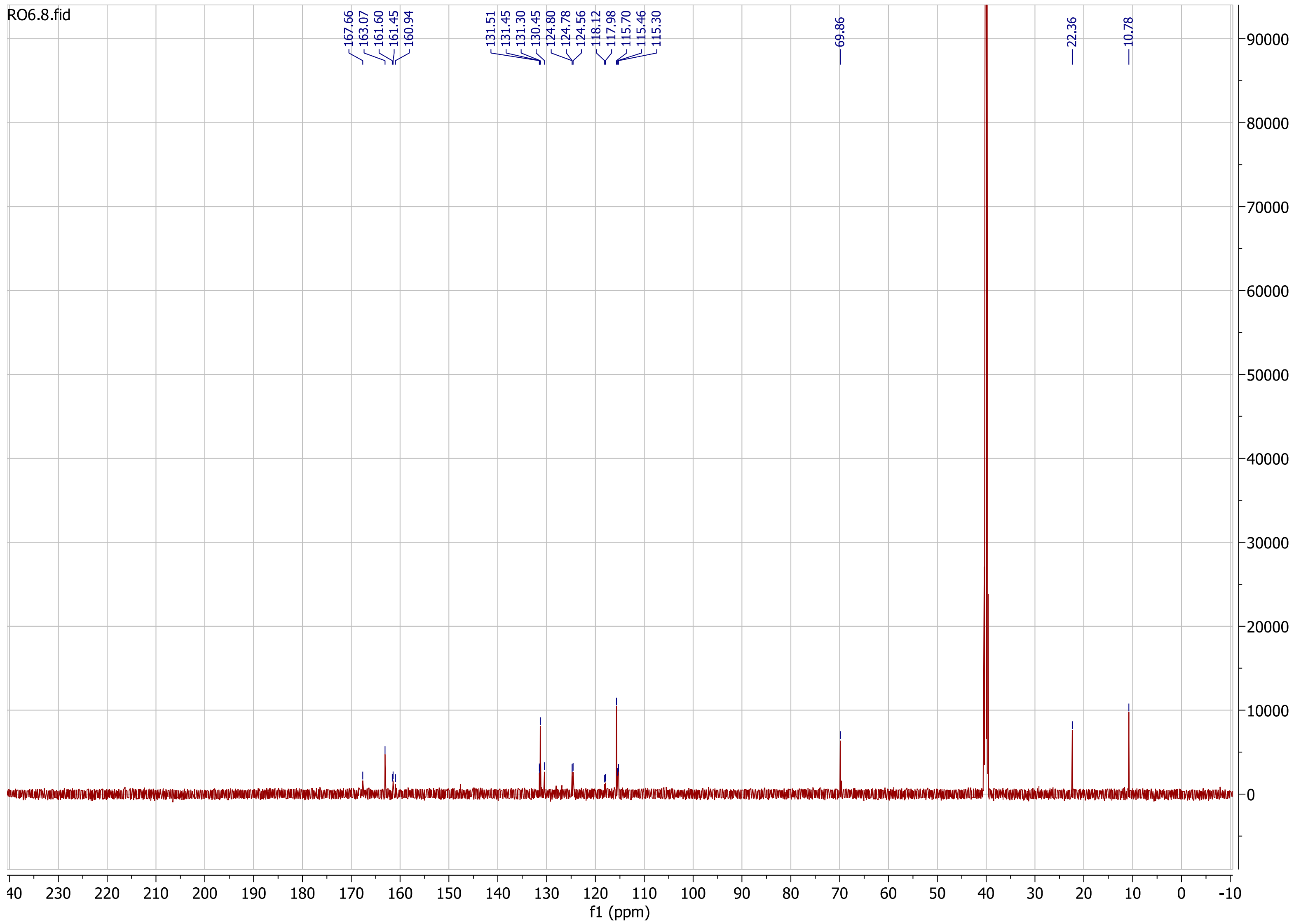

Figure S14. The ^{13}C NMR of compound RO6.

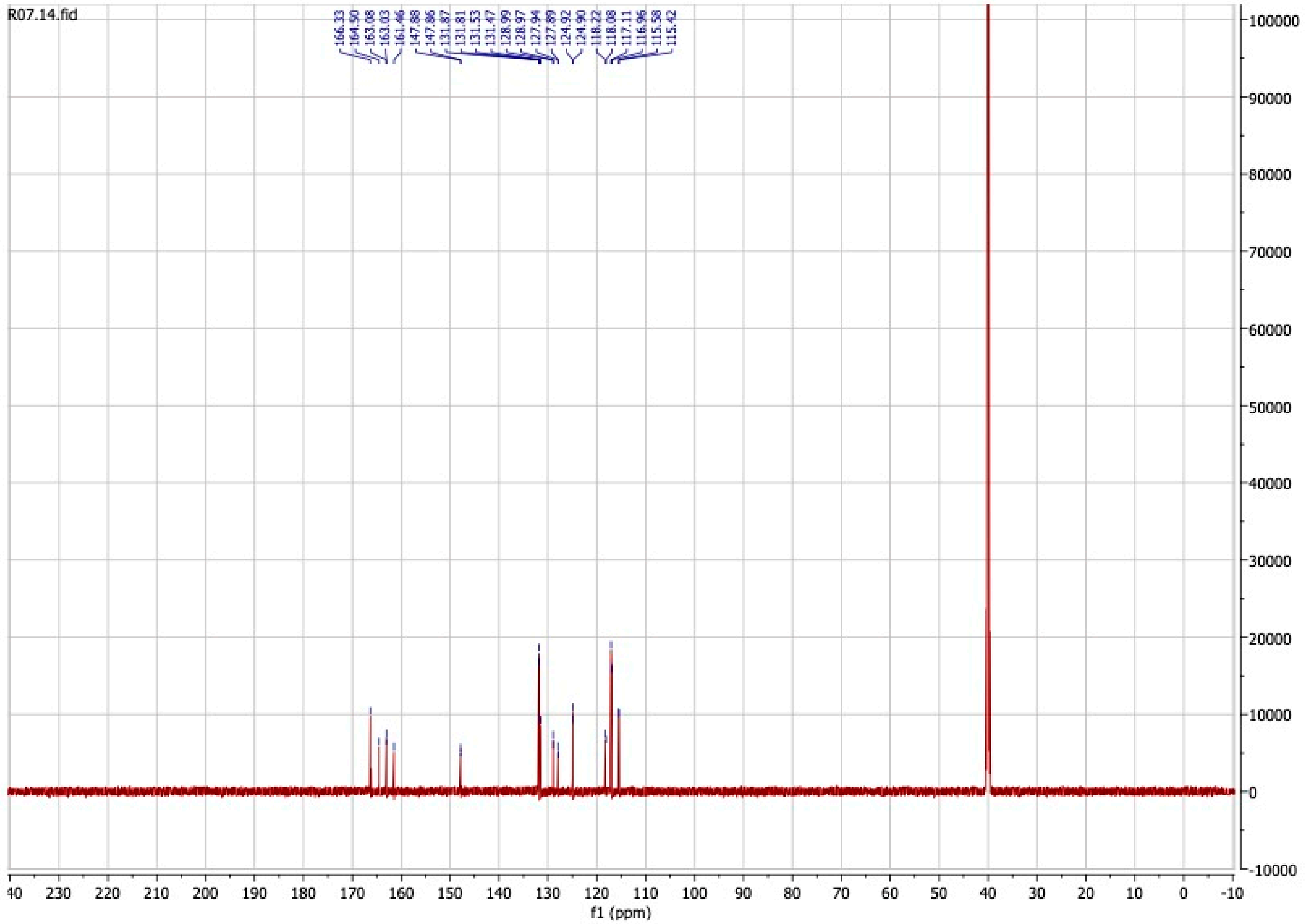

Figure S15. The ^{13}C NMR of compound RO7.

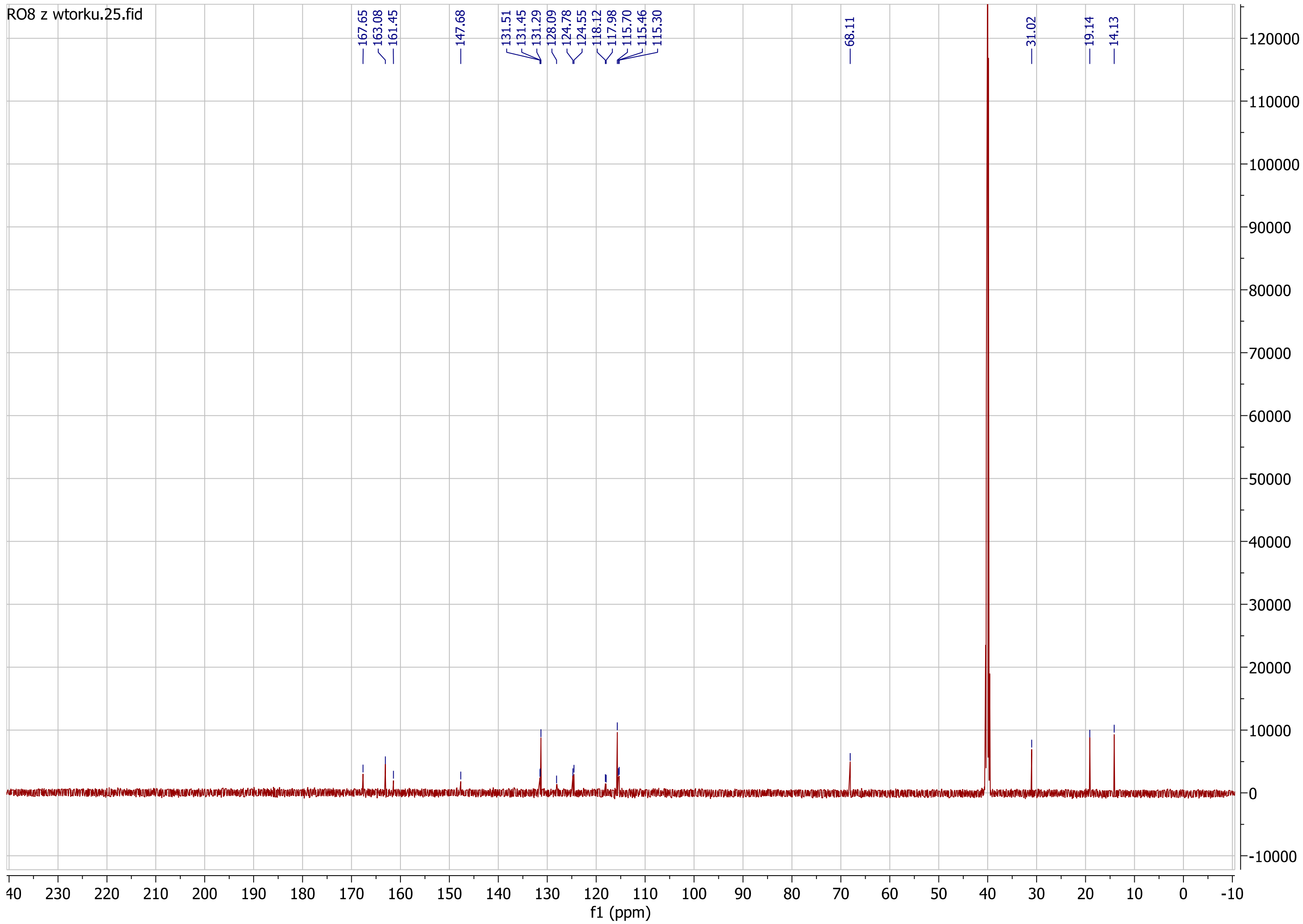

Figure S16. The ^{13}C NMR of compound RO8.

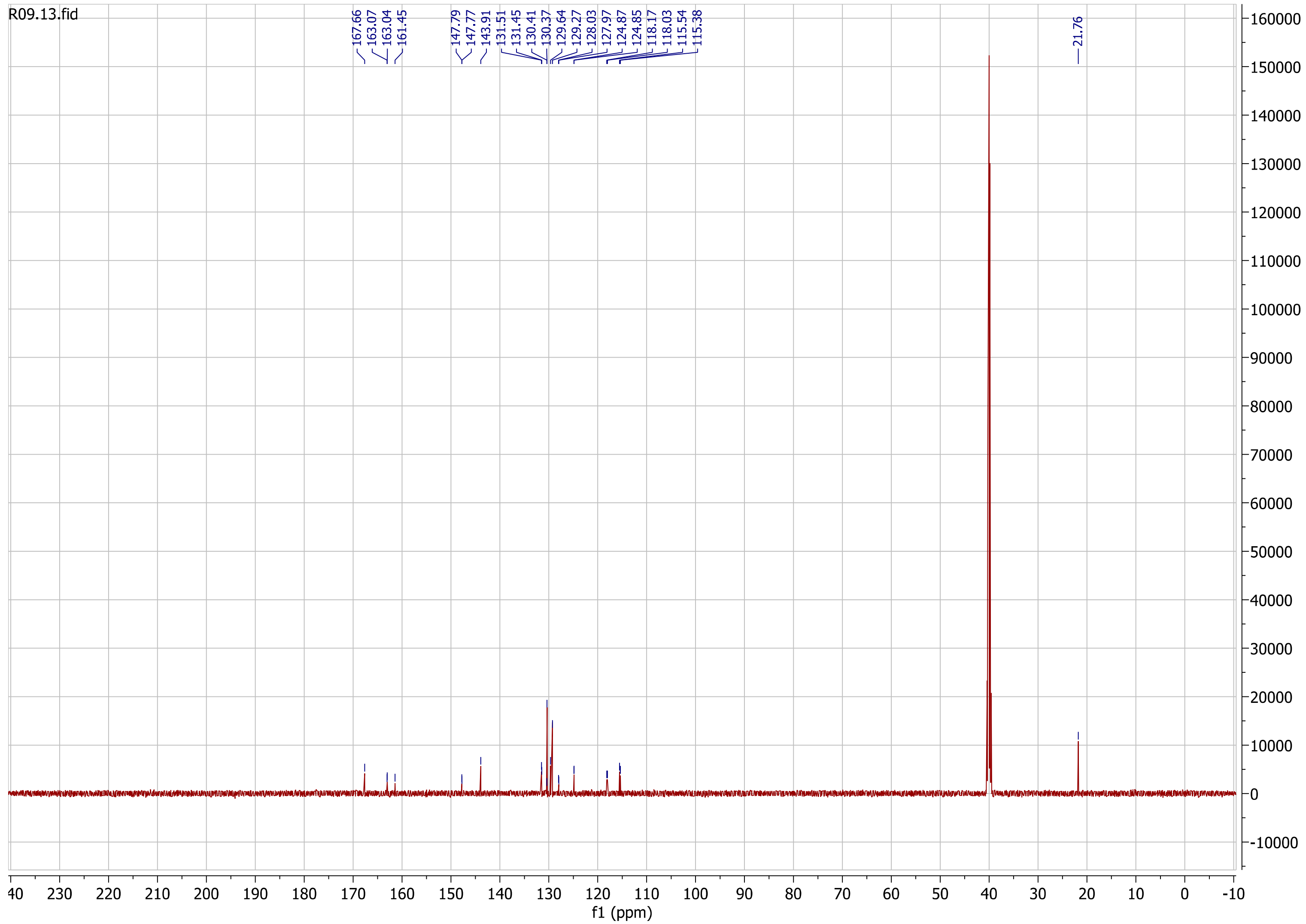

Figure S17. The ^{13}C NMR of compound RO9.

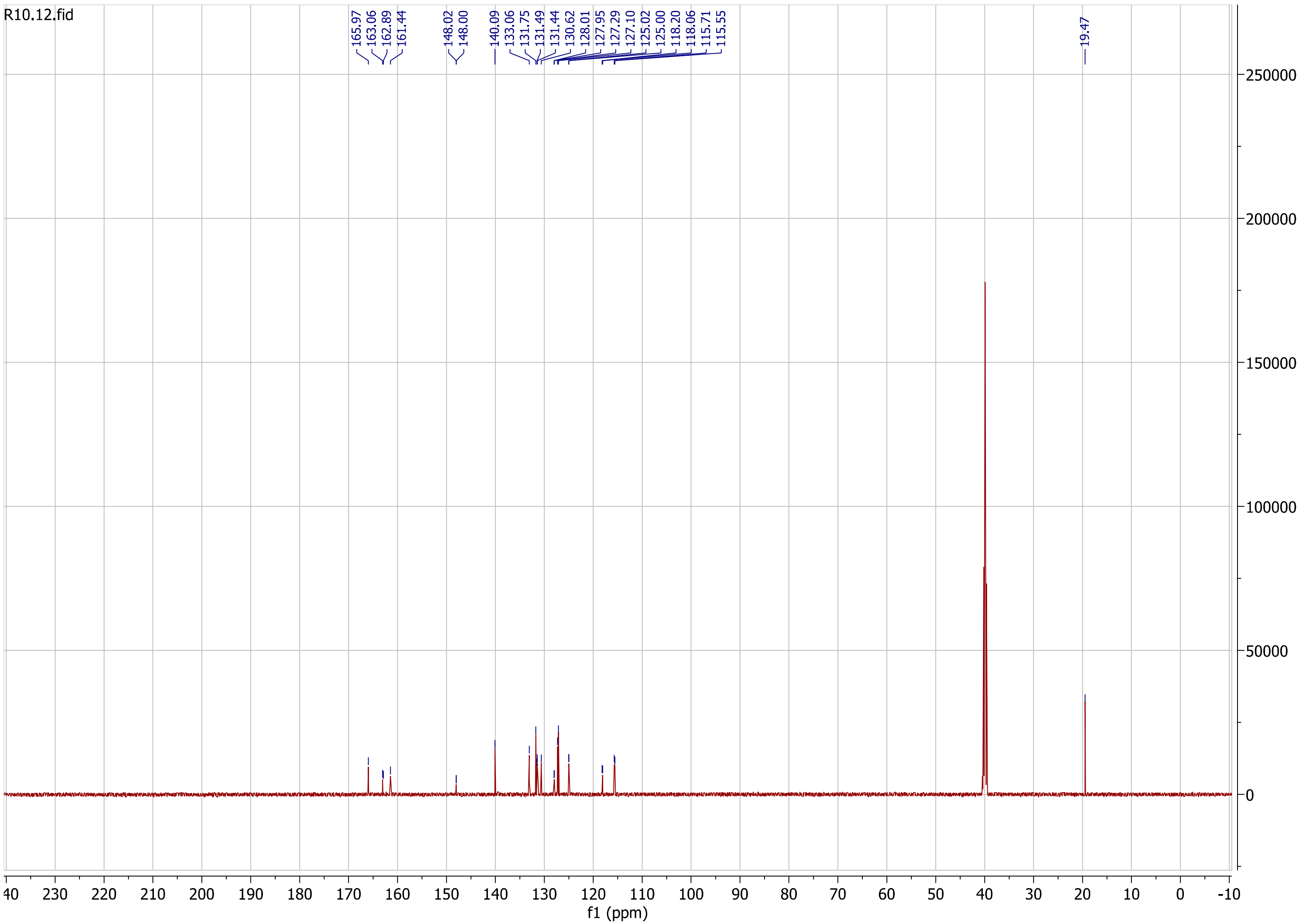

Figure S18. The ^{13}C NMR of compound RO10.

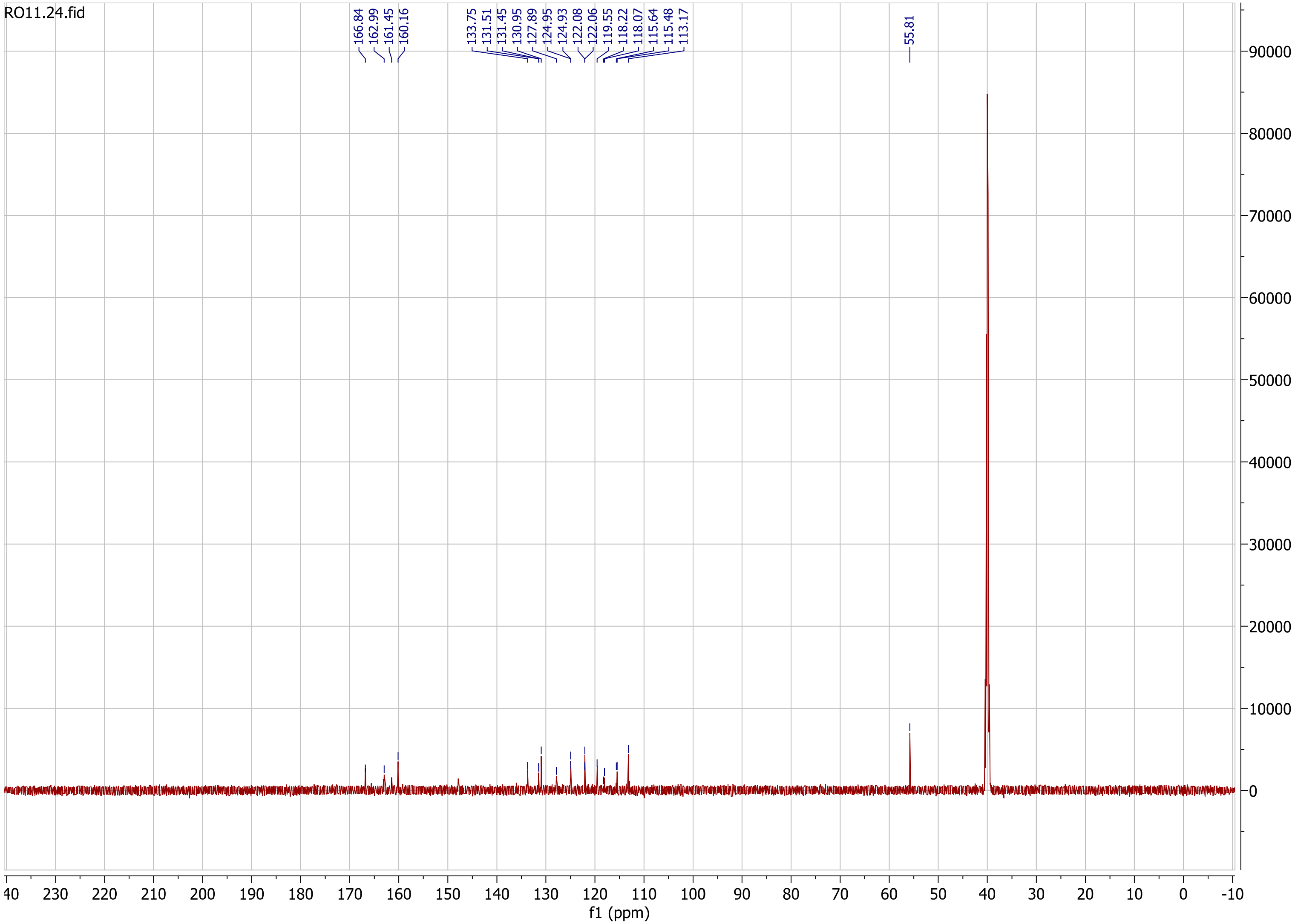

Figure S19. The ^{13}C NMR of compound RO11.

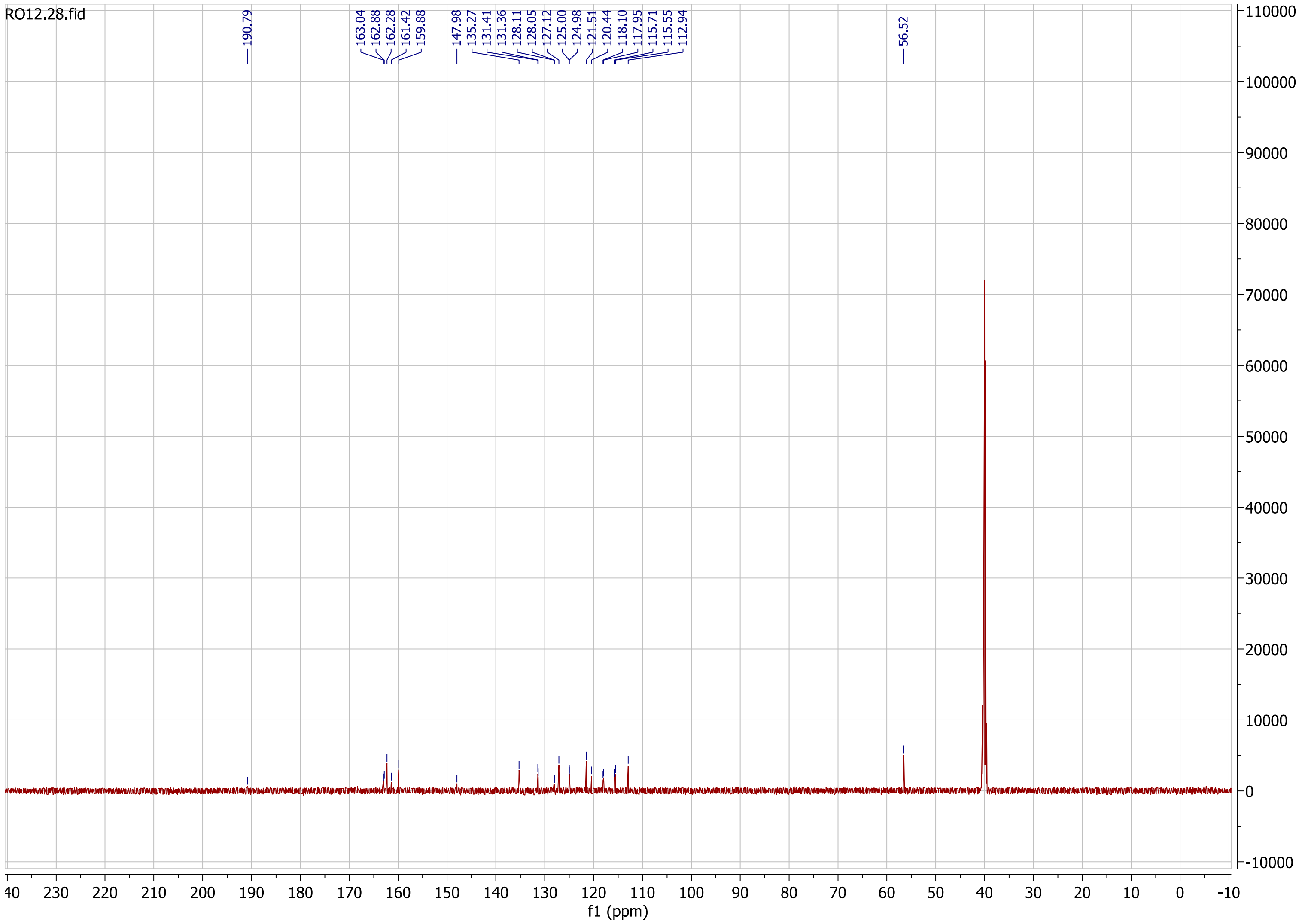

Figure S20. The ^{13}C NMR of compound RO12.
